# Spectroscopic and theoretical studies of fluorescence effects induced by the ESIPT process in a new derivative *2-Hydroxy-N-(2-phenylethyl)benzamide* – Study on the effects of pH and medium polarity changes

**DOI:** 10.1371/journal.pone.0229149

**Published:** 2020-02-25

**Authors:** Agnieszka Niemczynowicz, Iwona Budziak, Sławomir Kulesza, Andrzej Górecki, Marcin Makowski, Dariusz Karcz, Karolina Starzak, Bożena Gładyszewska, Janusz Podleśny, Agnieszka I. Piotrowicz-Cieślak, Arkadiusz Matwijczuk

**Affiliations:** 1 Department of Analysis and Differential Equations, Faculty of Mathematics and Computer Science, University of Warmia and Mazury, Olsztyn, Poland; 2 Department of Chemistry, University of Life Sciences in Lublin, Lublin, Poland; 3 University of Warmia and Mazury in Olsztyn, Faculty of Mathematics and Informatics, Chair of Relativistic Physics, Olsztyn, Poland; 4 Department of Physical Biochemistry, Faculty of Biochemistry, Biophysics and Biotechnology of the Jagiellonian University, Kraków, Poland; 5 Department of Theoretical Chemistry, Faculty of Chemistry, Jagiellonian University, Kraków, Poland; 6 Department of Analytical Chemistry (C1), Faculty of Chemical Engineering and Technology, Cracow University of Technology, Cracow, Poland; 7 Department of BioPhysics, University of Life Sciences in Lublin, Lublin, Poland; 8 Institute of Soil Science and Plant Cultivation—State Research Institute, Puławy, Poland; 9 Department of Plant Physiology, Genetics and Biotechnology, Faculty of Biology and Biotechnology, University of Warmia and Mazury in Olsztyn, Olsztyn, Poland; University of Lincoln, UNITED KINGDOM

## Abstract

The paper presents the results of studies conducted with the use of stationary and time-resolved fluorescence spectroscopy for the new derivative *2-Hydroxy-N-(2-phenylethyl)benzamide* (SAL-3) in aqueous solutions with various concentrations of hydrogen ions as well as in solvent mixtures (i.e. media with changing polarity/polarizability). For the compound selected for the study placed in aqueous solutions with varying concentrations of hydrogen ions, the fluorescence emission spectra revealed a single emission band within most of the pH range, however, at low pH (pH<3) a significant broadening (noticeable effect of dual fluorescence) and shifting of the band was observed. Whereas, for water and polar (protic) solvents, we observed a very interesting phenomenon of dual fluorescence never before reported for this particular group of analogues (with the specific substituent system). Based on the results of the experiments, it was observed that the presented effects may be related both with conformational effects (related to the possible positioning of the–OH group on the side of the carbonyl system, which facilitates the possibility of proton transfer) as well as, most importantly, the effects of excited state intramolecular proton transfer (ESIPT–Excited State Intramolecular Proton Transfer) related in this case with the necessary (new/previously unobserved in published literature) presence of ionic and non-ionic forms of the compound). Both the conducted quantum-mechanical [TD]DFT–Time-Dependent Density Functional Theory) calculations and excited state dipole moment change calculations for the analyzed molecule in solvents with varying pH confirmed the association between the observed fluorescence phenomena and the two aforementioned effects.

## 1. Introduction

Salicylic acid (SA) and its derivatives (phenylpropanoids) are commonly found in plants. Metabolically, their changes are related to the plant’s defensive reaction to biotic and abiotic stress [1]. The metabolism of phenylpropanoids shows high specificity and the respective paths of its synthesis are activated under very specific conditions, in specific phases of cell differentiation or under the influence of very specific environmental stimuli. Salicylic acid serves as the transmitter in plant defensive reactions, it can be synthesized based via two separate and unrelated synthesis paths taking advantage of different precursors: phenylalanine and the isochorismic path present in chloroplasts [2].

Phenylpropanoids are commonly used against various pathogens. Some derivatives of salicylic acid, such as *2-hydroxy-*N*-(arylalkyl)benzamides*, show antimicrobial activity against microscopic fungi and various bacterial strains, including mycobacteria [3], whereas several other compounds are capable of inhibiting the proliferation and vitality of human cancer cells [4]. The compound selected for the present study, *2-hydroxy-N-(2-phenylethyl)benzamide*, demonstrated 20% growth inhibition of *Mycospharella pinodes* species. *Mycosphaerella blight* caused by *Mycosphaerella pinodes* (Berk. et Blox. Vestergr) is one of the main diseases encountered in pea cultivations [5]. It occurs commonly in the fields of Europe, North America, Australia and New Zealand [6] and, on average, leads to a 10% decrease in yields, whereas in years when the temperature and humidity conditions are particularly unfavorable, yield losses can exceed 50% [5]. Limiting the incidence of the disease requires the use of adequate crop protection products. Hence, it is important to discover the photochemical/photophysical properties of *2-hydroxy-N-(2-phenylethyl)benzamide* in order to use it as a viable crop protection agent.

**Fig 1:**
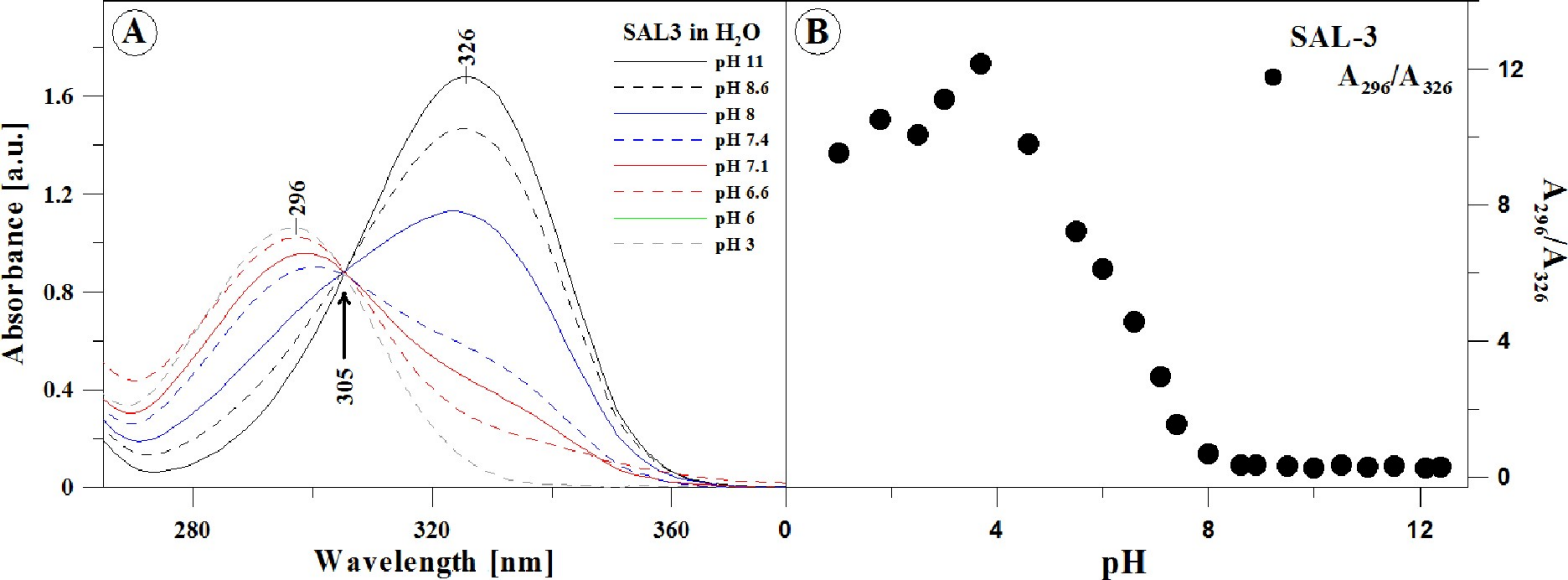
**Panel A:** Electronic absorption spectra of SAL-3 in aqueous solutions with various pH. The arrow marks the isosbestic point at the wavelength of ~305 nm. The figure presents spectra for pH: 3; 6; 6.6; 7.1; 7.4; 8; 8.6, and 11. The experiments were conducted at 23 ^o^C. **Panel B**: Correlation between the absorbance ratio at the wavelength of 296 to 326 nm relative and the pH of the aqueous solution, based on the spectra in Panel A.

Due to its considerable potential related to the aforementioned properties, *2-hydroxy-N-(2-phenylethyl)benzamide* (SAL-3 –Fig 1A–1C) was selected for our study on the mechanisms of molecular interactions. It was observed that the selected derivative of SA produces very interesting effects of dual fluorescence whose mechanism in the selected new derivatives has not been researched or associated with various other analogues already described in literature to date [7–9]. Identifying the exact mechanism of dual fluorescence in the selected analogue may prove significant to explaining the mechanisms of molecular interactions taking place in both itself and the entire group of new SA derivatives [10–13].

The aim of this study was to conduct spectroscopic and theoretical analyses of the molecular organization present in SAL-3 in aqueous solutions within the entire range of hydrogen ions’ concentrations as well as in selected organic solvents and in mixtures thereof (with water). In this context, the primary aim of the study was to identify the molecular mechanism and propose the theoretical model for the effects of dual fluorescence observed in the fluorescence emission spectra of this derivative. By employing spectroscopic methods such as: electronic absorption and fluorescence spectroscopy (including the RLS—Resonance Light Scattering) technique [14, 15], fluorescence lifetime measurements (TCSPC—Time-Correlated Single Photon Counting), supported by M. Kasha’s exciton splitting theory [16], [TD]DFT calculations, and determinations of the changes in excited state dipole moment changes, we were able to evidence the complexity of the physical processes responsible for the emergence of the dual fluorescence effect. The changes in dipole moments observed in the excited and ground states, as well as their respective ratios, confirm the possible presence of an additional electronic state related to the ESIPT process, whereas the quantum-mechanical calculations confirmed the possibility of the balanced presence of various ionic and non-ionic forms of the compound, which facilitates the possibility of dual fluorescence related to the ESIPT- phenomenon. It is noteworthy that previous literature reports for many analogues of this derivative have not suggested this path in the explanation of the aforementioned effects.

The effects related to the dual nature of fluorescence [17, 18] may be induced by changes in the solvent’s polarity, pH [17], temperature, or concentration of the analyzed compound [19, 20]. The most prevalent explanations offered for this type of effects include the emergence of intramolecular CT (charge transfer) states [19], particularly the twisted intramolecular charge transfer (TICT) [21, 22]. A characteristic feature of molecules showing this type of effects is the observed high excited state dipole moment and relatively fast rate of stabilization with the increase in the medium’s polarity. Another explanations can be made based on effects related to the presence of excimer systems [23, 24], the processes of excited-state intramolecular proton transfer (ESIPT) [25–29] or breaking Kasha’s rule for polyatomic molecules (Brancato et al. [30]). The most recent studies on the group of 1,3,4-thiadiazoles with the *2*,*4-dihydroxyphenyl* substituent, where the effects of dual fluorescence are induced by processes related to the phenomenon of molecular aggregation [31–33], which in turn lead to the emergence of CT processes in certain analogues [17–19].

Apparently this is not the case in the compound investigated in this work and the fluorescence effects observed more likely originate from the excited-state intramolecular proton transfer (ESIPT) [27, 34, 35]. It is also worth-emphasizing that the compound studied in this work is characterized by conformational flexibility and can adopt two conformations, which may either allow or suppress the proton transfer process. This structural feature may significantly contribute to the fluorescence effects observed. The last important factor relates to the possibility (depending on the polarity/pH of the medium) of the emergence of ionic and non-ionic forms which most precisely correlate with the observed fluorescence effects, and which have not yet been observed for any derivative described in worldwide literature.

Studies conducted with the use of fluorescence spectroscopy, both stationary and time-resolved, on the SAL-3 compound, allowed us to observe the effect of dual fluorescence in aqueous solutions complemented with various proportions of a protic solvent (as well as in protic solvents themselves). In water solutions, with changing concentrations of hydrogen ions, we registered only a single fluorescence emission band of varying intensity depending on the changes in pH. Only at very low pH levels were we able to observe a shift of the main emission band and its broadening on the short wavelength side (a relatively clearly visible effect indicating the possibility of a second emission band).

Uncovering the mechanism of the compound’s organization in media of varying pH may significantly contribute to the determination of its potential pharmacological applications as well as to future model studies aimed at identifying its specific molecular properties. Furthermore, the compound (and the entire group of analogues) may, due to its high quantum efficiency, also find applications as a fluorescence probe sensitive to changes in the pH or polarity of the medium.

## 2. Materials and methods

### 2.1. Materials

Molar volumes were calculated with ChemSketch software (2015). NMR spectra were recorded with an NMR spectrometer Varian Unity Plus 500 MHz. Characteristics of the investigated compound are presented in Table 1.

**Table 1:** Characteristics of the investigated compound (Fig 2).

Compound: Systematic name (common name)	Molecular weight [g^.^mol^-1^]	Melting point [^o^C]	Molar volume [cm^3.^mol^-1^]	pK_a_ (temp. [^o^C])	logP_oct_	logD (pH)	CAS registry number
2-Hydroxy-N-(2-phenylethyl)benzamide	137.14	140.2–141.2	106.5 ± 3.0	8.37 (20)	1.06	1.06 (5.1)	65-45-2

#### 2-Hydroxy-*N*-(2-phenylethyl) benzamide

A mixture of 15.21 g (0.1 mol) of methyl salicylate and 12.12 g (0.1 mol) of phenethylamine was left in a stoppered bottle for two weeks, occasionally shaken, at room temperature. Next, the syrupy liquid was poured into diluted hydrochloric acid. The precipitated mass was filtered and rinsed several times with cold water. After air-drying, the yield was 19.53 g (80.2%). For further studies, the product was additionally recrystallized two times from a mixture of chloroform-methanol (1:1) and dried in vacuum for 3 h at room temperature. The physicochemical characteristics were as follows: m.p. 93.6–94.7 ^o^C; ^1^H-NMR (500 MHz, CDCl_3_, δ): 11.97 (bs, 1H, -COOH), [7.33–7.42 (m, 3H), 7.23–7.31 (m, 3H), 7.21 (dd, 1H, *J*_1_ = 8.1 Hz, *J*_2_ = 1.5 Hz), 6.99 (d, 1H, *J* = 8.3 Hz), 6.81 (t, 1H, *J* = 7.6 Hz) (phenyl)], 6.39 (bs, 1H, -NH-), 3.72 (q, 2H, *J* = 6.7 Hz), 2.95 (t, 2H, *J* = 6.9 Hz); ^13^C-NMR (125 MHz, CDCl_3_, δ): 169.87 (-COOH), [161.46, 138.40, 134.14, 128.78, 128.72, 126,73, 125.12, 118.61, 118.55, 114.18 (phenyl)], [40.70, 35.47 (aliphatic)].

### 2.2. Methods

#### 2.2.1 AFM measurements

Atomic Force Microscopy (AFM) relies on the mapping surface geometry of solids determined at micro- and nano-levels by sampling the forces occurring due to the interaction with a conical tip with an atomically-sharp apex. The probe is mounted on an elastic cantilever, the bending of which can be detected by measuring laser beam reflection.

Geometrical structure plays a major role in tribology, contact mechanics and dynamic processes on the surface of films at various levels of complexity [36, 37]. In this aspect, AFM serves as a tool for characterizing the spatial geometry of texturized films. In the current study, the AFM method was employed to image the geometrical structure of SAL-3 crystal samples along with the investigation of their nanomechanical properties (as additional properties of this compound). AFM measurements were carried out using a Multimode 8 instrument with a Nanoscope V controller (Bruker) and NSG11 scanning tips (NT-MDT) in air, under ambient conditions. The estimated tip radius was 5 nm and the cantilever force constant was 5 N/m. The scan area varied between 1 and 5 μm with 256 steps along each scan axis. The AFM data were recorded in the Peak Force Quantitative Nanomechanical Mapping (PF-QNM) mode that maps the surface topography together with selected mechanical properties such as: reduced Young’s modulus, adhesion forces, and sample deformation. Here, the maximum repulsive force between the tip and the surface was set to 2 nN.

#### 2.2.2 Electronic absorption and fluorescence spectra

The electronic absorption spectra of SAL3 were recorded on a double-beam UV-Vis spectrophotometer Cary 300 Bio (Varian) equipped with a thermostatted tray holder with a 6×6 multi-cell Peltier block. The temperature was controlled with a thermocouple probe (Cary Series II from Varian) placed directly in the sample.

Fluorescence excitation, emission, and synchronous spectra were recorded with a Cary Eclipse spectrofluorometer (Varian) at 22 ^o^C. Fluorescence spectra were recorded at the resolution of 0.5 nm and corrected for the lamp and photomultiplier spectral characteristics. Resonance light scattering (RLS) measurements were performed as described by Pasternack and Collings. The excitation and emission monochromators of the spectrofluorometer were scanned synchronously (0.0 nm interval between the excitation and emission wavelengths); the slits were set to obtain a spectral resolution of 1.5 nm. The spectral analysis was performed with the use of Grams/AI 8.0 software (Thermo Electron Corporation).

#### 2.2.3 Time-correlated single photon counting (TCSPC)

Time-correlated single photon counting (TCSPC) measurements were performed using a FluoroCube fluorimeter (Horiba, France). The samples were excited with a pulsed Nano- LED diode at 294 nm (pulse duration of 700 ps) operated with 1 MHz repetition. To avoid pulse pile-up, the power of the pulses was adjusted to an appropriate level using a neutral gradient filter. Fluorescence emission was recorded using a TBX-04 picosecond detector (IBH, JobinYvon, UK). The Data Station and DAS6 software (JobinYvon (IBH, UK)) was used for data acquisition and signal analysis. All fluorescence decays were measured in a 10 x 10 mm quartz tray using an emitter cut-off filter 408 nm. The excitation profiles required for the simplified analysis were measured without the emitter filters on a light scattering tray. All measurements were performed at 20 ^o^C in various solvents. Each case of fluorescence decay was analyzed with the multi-exponential model shown in the equation:

It=∑iαiexp(−tτi) (1)

where α_i_ and τ_i_ are the pre-exponential factor and the decay time of component *i*, respectively.

The best-fitted parameters were obtained by minimizing the reduced χ^2^ value as well as through residual distribution of the experimental data. The fractional contribution (*f*_*i*_) of each decay time and the average lifetime of fluorescence decay (<τ>) were calculated with the following equations:

$$f_{i}=\frac{\alpha_{i}\tau_{i}}{\sum_{j}\alpha_{j}\tau_{j}}$$ (2)

$$\langle \tau \rangle =\sum_{i}f_{i}\tau_{i}$$ (3)

#### 2.2.4 DFT calculations

A Gaussian 09 package [38], using B3LYP exchange-correlation functional [39], aug-cc-pVDZ basis set [40] and very dense grids, was applied in the DFT calculations. Dispersion effects were accounted for using Grimme’s D3 semi-empirical treatment with the Becke-Johnson damping scheme [41]. The solvent effects were treated by Polarizable Continuum Model (PCM) [42]. Charged species were modelled using Na^+^ and Cl^-^ as counterions. The excited state treatment was carried out using the standard random-phase approximation (RPA) approach to TD DFT formalism [43][A6]. The influence of the solvent on energetics was determined according to the linear response approximation.

#### 2.2.5 Estimation of the dipole moments

In the presented study, the ground and excited-state dipole moment of the *2-hydroxy-N-(2-phenylethyl)benzamide* (SAL-3) molecule was estimated based on two methods based on the influence of the internal electric field (solvatochromism). The influence of the solvents on the locations of the maxima of absorption and fluorescence spectra is used to evaluate the excited-state dipole moments of the molecules.

*Method 1*. Based on the papers by Bakshiev and Kawski-Chamma-Viallet [44], the change in the dipole moment can be calculated from the following two Eqs (4 and 5):

$${\overset{¯}{v}}_{a}-{\overset{¯}{v}}_{f}=m_{1}F_{1}\left(\varepsilon ,n\right)+cste$$ (4)

$$\frac{{\overset{¯}{v}}_{a}+{\overset{¯}{v}}_{f}}{2}=m_{2}F_{2}\left(\varepsilon ,n\right)+cste$$ (5)

Where ${\bar{v}}_{a}$ and ${\bar{v}}_{f}$ are the absorption and fluorescence maxima expressed in cm^-1^. The expressions *F*_1_(*ε*,*n*) (Baksviev’s polarity function) and *F*_2_(*ε*,*n*) (Kawski-Chamma-Viallet function) for solvents are developed as Eqs (6 and 7).

$$F_{1}\left(\varepsilon ,n\right)=\frac{2n^{2}+1}{n^{2}+2}\cdot \left(\frac{\varepsilon -1}{\varepsilon +2}-\frac{n^{2}-1}{n^{2}+2}\right)$$ (6)

$$F_{1}\left(\varepsilon ,n\right)=\frac{2n^{2}+1}{2\left(n^{2}+2\right)}\cdot \left(\frac{\varepsilon -1}{\varepsilon +2}-\frac{n^{2}-1}{n^{2}+2}\right)+\frac{\left(n^{4}-1\right)}{2{\left(n^{2}+2\right)}^{2}}$$ (7)

Where n is the light refraction index and ε is the solvent’s dielectric constant. The dipole moment change is calculated from the locations of absorbance and fluorescence maxima for the given molecule in various solvents. Assuming that the symmetry of the analyzed molecule remains unchanged following the electronic transition, and that the ground and excited-state dipole moments are parallel, we obtain the following Eqs (8 and 9):

$$\mu_{g}=\frac{\left|m_{1}+m_{2}\right|}{2}\cdot {\left(\frac{hca_{0}^{3}}{2m_{1}}\right)}^{1/2}$$ (8)

$$\mu_{e}=\frac{\left|m_{1}-m_{2}\right|}{2}\cdot {\left(\frac{hca_{0}^{3}}{2m_{1}}\right)}^{1/2}$$ (9)

Where μ_g_ and μ_e_ are the ground and exited-state dipole moments, respectively, h is Planck’s constant, c is the velocity of light, and *a*_0_ is the radius of the molecule’s Onsager cavity. The cavity radius for a dissolved substance – *a*_0_ - is calculated from Suppan’s equation [45] (10):

$$a_{0}={\left(3M/4\pi \delta N\right)}^{1/3}$$ (10)

Where δ is the density of the dissolved substance, M is the molar mass, and N is the Avogadro constant. The ration of the excited-state dipole moment to the ground-state dipole moment can be expressed as (11):

$$\frac{\mu_{e}}{\mu_{g}}=\frac{\left|m_{1}-m_{2}\right|}{\left|m_{1}+m_{2}\right|}$$ (11)

Where m_1_ and m_2_ are the respective slopes of straight lines obtained from the graphs representing the Stokes shift relative to the function of solvent polarity *F*_1_(*ε*,*n*) and $({\bar{v}}_{a}+{\bar{v}}_{f})/2$ relative to the function of *F*_2_(*ε*,*n*). The change in the molecule’s dipole moment is determined on the basis of the difference between the excited-state dipole moment and the ground-state dipole moment (Eq 12):

$$\Delta \mu =\mu_{e}-\mu_{g}$$ (12)

*Method 2*. The second method, first proposed by Reichardt [46], utilizes a scale describing the microscopic polarity of solvents $E_{T}^{N}$ which is an assessment based on the solvatochromic effect present in the betaine pigment. The polarization dependence and presence of hydrogen bonds in solvents are correlated with the polarity scale $E_{T}^{N}$. The theoretical basis for the correlation between the spectral shift and the $E_{T}^{N}$ scale was provided by Ravi et al. [45]. The $E_{T}^{N}$ value is defined by Eq (13), where water ($E_{T}^{N}=1$) and tetramethylsilane ($E_{T}^{N}=0$) are used as the reference solvents:

$$E_{T}^{N}=\frac{E_{T}\left(solvent\right)-E_{T}\left(TMS\right)}{E_{T}\left(water\right)-E_{T}\left(TMS\right)}=\frac{E_{T}\left(solvent\right)-30.7}{32.4}$$ (13)

Finally, the molecular dipole moment change Δμ can be described as (14):

$$\Delta \mu =\mu_{e}-\mu_{g}=\sqrt{\frac{m\cdot 81}{11307.6\cdot {\left(\frac{6.2}{a_{0}}\right)}^{3}}}$$ (14)

Where m is the slope of the straight line obtained from the graph of the Stokes shift relative to the $E_{T}^{N}$ parameter.

## 3. Results and discussion

### 3.1 AFM study

**Fig 2:**
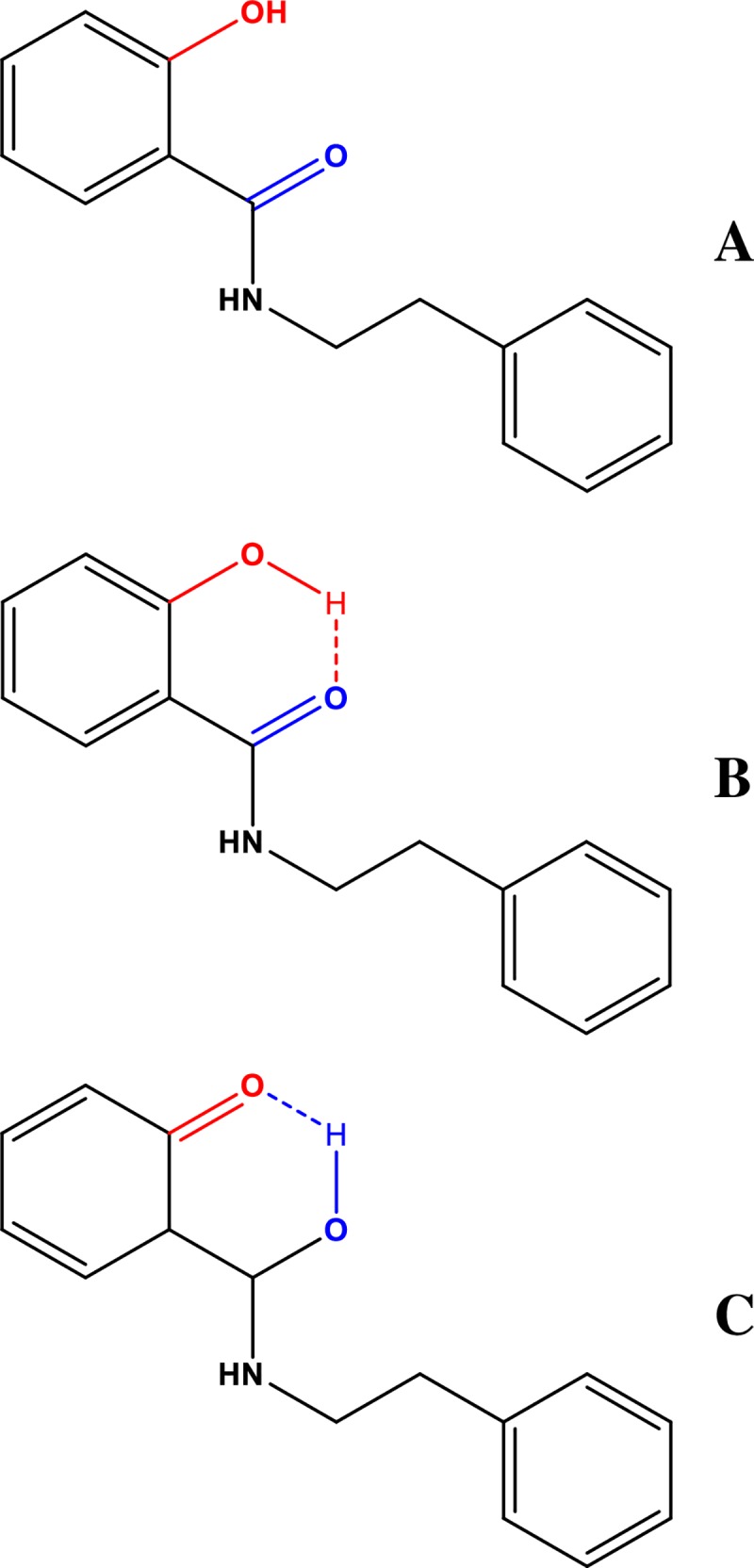
Chemical structure of the SAL-3 molecule (A–non-ionized form, B–form with an H-bond between the–OH and C = O group, C–form after proton transfer between the–OH and C = O group, in this case a carbonyl group is formed and an–OH group emerges where the carbonyl group used to be).

The structure of the analogue selected for the study is presented in Fig 2A–2C.

As can be observed, the SAL-3 molecule is capable of forming an intramolecular hydrogen bond between the–OH group and the adjacent carbonyl group. Fig 2B and 2C also evidence the molecule’s capacity for proton transfer. Furthermore, the powder of the compound itself was analyzed with the use of AFM measurements and the results were provided in S1–S5 Figs enclosed in Supplementary Materials. The surface was found to be strongly layered with flat, irregular terraces and sharp steps, each around 1 nm in hight, as best seen in S2 Fig. Apart from that the above, structural inhomogeneities in the form of the irregular layers of foreign material—2 nm thick and a few hundred nanometers in size (“A” marks)—can also be seen. In addition, a large area of circular particles from 10 up to 100 nm in diameter can be spotted (“B” marks). These particles are randomly distributed over the surface of the crystals with no apparent alignment pattern, which excludes both molecular agglomeration and edge attraction phenomena, and consequently surface diffusion processes, from further analysis.

### 3.2 Spectroscopic studies

**Fig 3:**
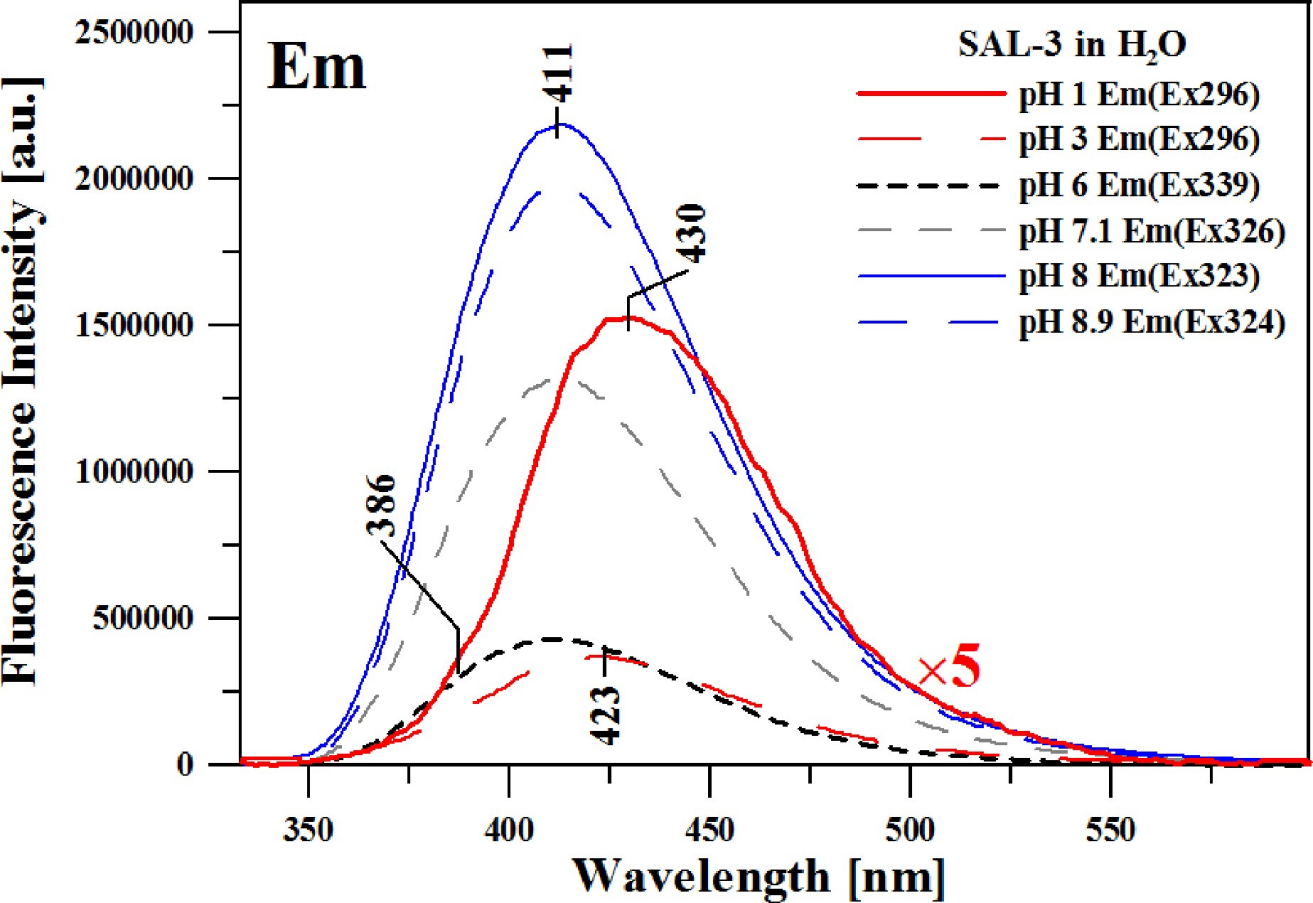
Fluorescence emission spectra for SAL-3 in aqueous solutions with various pH. The figure presents the results for pH: 1; 6.5; 7.1; 8; 8.9; and 11. The spectra were obtained at 23°C in the spectral range from 305 to 600 nm.

Fig 1, Panel A presents the results of absorption spectra measurements performed for SAL-3 with the use of electronic absorption spectroscopy in the full range of hydrogen ion concentrations. The results indicate very clear changes in the shapes of the spectra, particularly in the region important for physiological values. For better clarity of the presentation, the spectra obtained for the analyzed compound at pH levels of: 3, 6, 6.6, 7.1, 8, 8.6, and 11 are presented. As can be observed in Fig 1, Panel A, dissociation of the–OH group from the hydroxybenzene ring in the *ortho* position (Fig 2) causes a hypsochromic spectral shift by 996 cm^-1^ (9 nm) at pH 1 and bathochromic shift by 2112 cm^-1^ (21 nm) in the compound’s spectra at pH 12, relative to the spectra observed in polar (protic) solvents. Furthermore, in the case of the analogue selected for the study, the process of ionization can be accompanied by slight (but noticeable) processes of aggregation [16]. At approx. pH 7 (in Fig 1, Panel A, clearly for spectra at pH 7.1 or pH 7.4) there is a clear widening of the SAL-3 absorption bands (dashed blue and solid red lines), indicating the possible presence of spectral forms other than monomeric [16]. In the case of the compound’s spectra at low pH levels (e.g. pH 3, below pH 6), the spectrum absorbance is decreasing, moreover the band on the longwave side of the spectrum disappears (which in the analyzed case suggests, based on the [TD]DFT calculations, more likely the effects of various ionic forms of the compound as opposed to aggregation effects), which can indicate the significant prevalence of monomeric SAL-3 forms emerging in the given ionic form in the medium in question (see Panel A in Fig 1). Observed broadening of the bands at pH 7 compared to the respective spectra in the solvents shows the impact of heterogeneity of the system. The effects indicate (as confirmed in the [TD]DFT calculations) the occurrence of different ionic forms of the compound depending on the pH of the medium. Furthermore, we can also observe a clearly defined isosbestic point at the wavelength of 305 nm, which may indicate that the transition between the respective ionic (and non-ionic) forms of the compound is very fluid (which is further corroborated by the results of fluorescence spectra measurements). At pH levels such as 7.1 or 7.4 (as well as others not presented but within the same pH range) we can observe the aforementioned strong widening of the band on the longwave side with the maximum at approx. 326 nm, which suggests the prevalence of a specific ionic form (with the–O^-^ group) whose content remains at a probably continuously (slightly) increasing level with the increasing pH. This also indicates the prevalence of ionized forms of the SAL-3 molecule, the spectra where the widening disappears correlate with the prevalence of non-ionized forms present mostly as monomers (as will be further discussed in the text and which has a strong impact on fluorescence effects, Fig 3). It is also noteworthy that at low pH levels, protonation of the carbonyl group is also possible with the associated additional ionic form. Although not observed in the absorption spectra, the possible evidence of its presence was noted in the fluorescence spectra.

Panel B in Fig 1 presents the relationship between the absorbance maximum at 296 nm (prevalence of the monomeric form–non-ionized/or ionized in the carbonyl group) to 326 nm (prevalence of the associated form–and ionized in pH higher than physiological, with the–O^-^ group) for SAL-3 depending on the pH value of the analyzed aqueous solution. We can clearly observe that the level of monomerization (with the prevalence of the form containing an ionized–O^-^ group) for the selected analogue is the highest in high pH (to approx. pH 8), generally higher than physiological. Next, with the decreasing pH, within the range of 8 to 4.5, we observe the presence of both ionized and non-ionized forms, with the decreasing participation of the former. Meanwhile, at pH below 4, we once again observe the prevalence of ionized forms, and at very low pH levels (1, 2, or 3), there is the possibility of protonation also of the carbonyl group, emergence of an additional ionic form, which is not, however, seen in the absorption or fluorescence emission spectra. It should be emphasized that the most significant changes of the discussed ratio in the analyzed analogue are observed primarily within the physiological pH range, as can be clearly seen in the absorption spectra presented in Fig 1, Panel A.

The subsequent stage of the study entailed an analysis of the SAL-3 analogue with the use of fluorescence spectroscopy. One should point out the very interesting fluorescence effects illustrated in Fig 3.

It presents the fluorescence emission spectra for SAL-3, corresponding to the absorption spectra in Fig 1 (Panel A), relative to changes in the pH of the aqueous solution (the results are presented, respectively, for pH 1, 3, 6, 7.1, 8, and 8.9 –similarly to the absorption spectra in Fig 1, Panel A). The excitation wavelength for all the analyzed samples corresponded to the respective absorption spectra in Fig 1 (Panel A). As can be observed in the case of aqueous solutions with pH levels lower than physiological condition, the study revealed a clear shift of the emission band maximum to 430 nm and the emergence of a clear band widening on the shortwave side, with the maximum at approx. 386 nm (Fig 3). In the case of SAL-3, the effect is clearly present within the pH range from 1 to ~6. Above pH 6, we observe only a single fluorescence band with the maximum shifted to approx. 411 nm (also with significantly higher intensity). After longwave excitation (corresponding to the ionized forms with the–O^-^ group) with the maximum at approx. 326 nm for the samples in pH lower than 6, we observe only a single fluorescence emission band with the maximum at approx. 411 nm. The effect clearly indicates the possible coexistence of ionized and non-ionized forms of the molecule in the solution.

### 3.3 RLS study

**Fig 4:**
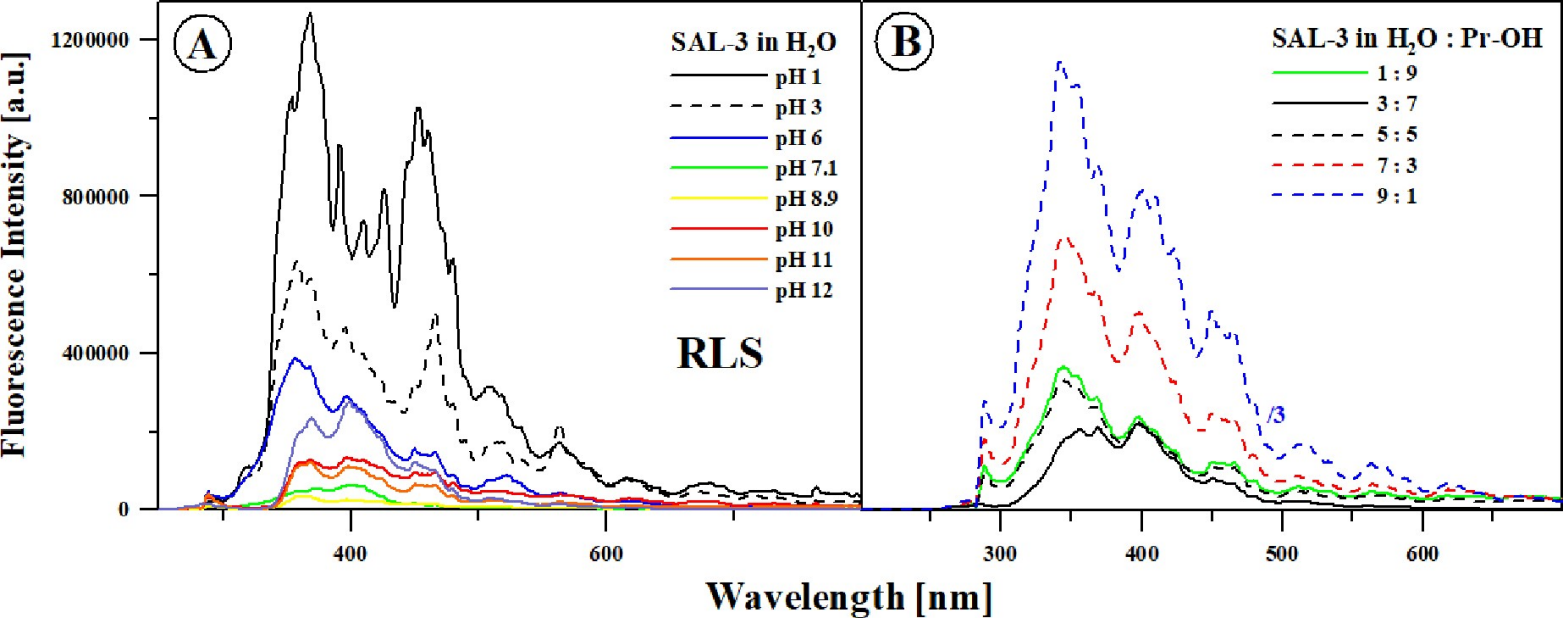
**Panel A:** Resonance light scattering (RLS) spectra for selected values of aqueous solution pH, analogically to the previous experiments. **Panel B:** RLS spectra for selected mixtures of H_2_O and propan-2-ol.

In order to determine the impact of the coexistence of various ionic forms of the analyzed compound on the observed fluorescence effect, we performed decomposition of a selected absorption spectrum (e.g. for pH 7.4 in S6 Fig in SM) and analysis (only qualitative) of its respective constituents, we also analyzed the RLS spectra for SAL-3 within the entire range of hydrogen ion concentrations (Fig 4A).

As can be observed in S6 Fig presenting the decomposition of the absorption spectrum of SAL-3 in pH 7.4, the bands were resolved into their respective constituents, with the ones with the maxima at approx. 296 nm (at this pH) and 326 nm (solid red and green lines) identified as the most significant contributors. It is clearly apparent that the higher the contribution of the constituent corresponding to the longwave part of the spectrum, the more quickly we can observe quenching of the effect of emission spectra widening on in the shortwave side in the fluorescence emission spectra, accompanied by a shift of the emission band to approx. 411 nm. The above effects confirm the earlier assumption that in order for the effect of dual fluorescence to be observed in the case of SAL-3, the additional condition of a relatively low concentration of the analyzed compound must be met, while aggregation effects are more likely to quench rather than induce it. The key question remains, however, whether the molecule is ionized or non-ionized, as this determines the emergence of the specific effects related to the widening of the fluorescence emission bands. In the example decomposition of the absorption bands, we can also observe the necessity of the presence (in order to obtain a good decomposition) of additional bands–such as the one with the maximum at approx. 285 nm. However, the emission from this band does not cause any clear changes in the emission spectra.

This fact, i.e. the coexistence of ionic and non-ionic forms, was further corroborated by the performed RLS [14] spectra measurements conducted in aqueous solutions at various concentrations of hydrogen ions, as already discussed above (Fig 4A). However, their presence was usually associated with the possibility of chromophoric aggregation of compounds present in the solution [14]. But as can be observed in Fig 4A, the RLS bands for the respective pH ranges vary in intensity and shape. They appear for various pH ranges, which suggests that the aggregation (chromophoric) slightly evidenced in the electronic absorption spectra has no significant impact on the observed effect of dual fluorescence, and its influence is mainly related to quenching the same. At the same time, the observed oscillatory structure of the RLS bands may evidence the potential presence of different forms: ionic and non-ionic, depending on the pH, whose relative prevalence may fluctuate [14] in various pH ranges, and therefore change the character of their mutual interaction. It is noteworthy that for pH levels of ~6 and lower, there is an additional broadening (very intensive) of the RLS bands, which indicates the possible presence of additional ionized forms (described above) at low pH levels, which could explain the additional effects observed in the fluorescence emission spectra at low pH.

**Fig 5:**
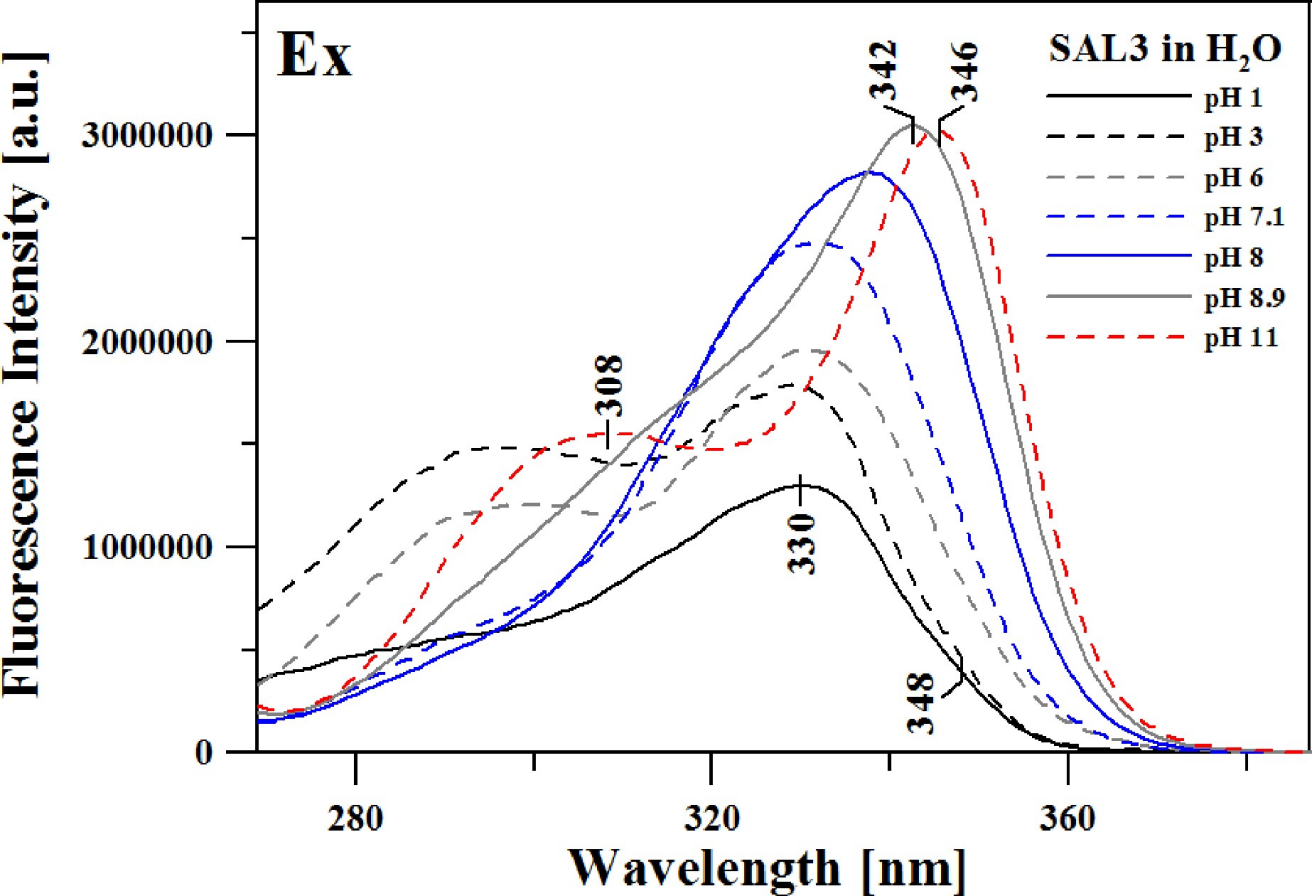
Fluorescence excitation spectra for selected pH of the aqueous solution. Excitation emission was set at the respective maximum of the longwave fluorescence emission spectra. The spectra were obtained at 23°C in the spectral range from 270 to 390 nm.

Fig 5 presents fluorescence excitation spectra corresponding to the emission spectra shown in Fig 3.

In this case, the excitation wavelength corresponded to the first or second (in the context of bands widening on the shortwave side of the emission spectra) maximum observed in the fluorescence emission spectra. The fluorescence excitation spectra, due to their selectiveness, reveal various spectral forms originating from, in our case, e.g.: iodized or non-ionized forms. This fact is confirmed by the aforementioned deconvolution analysis performed on the absorption spectra, as well as (partially) the RLS spectra. In this case we are dealing with bands originating from the ionized or non-ionized form of the SAL-3 molecule. As clearly indicated by Fig 5, at pH levels higher than ~6, one can observe a significantly higher intensity of the band on the longwave side–due to the prevalence of ionized forms observed at high pH, as well as the possible presence of an additional ionic form at low pH levels (as already discussed above). Meanwhile, at pH lower than mentioned above, a band maximum emerges which corresponds to the maximum of the main absorption band with the maximum at approx. 300 nm. The band is characteristic of the non-ionized form.

### 3.4 Spectroscopic effects in mixed solvents

**Fig 6:**
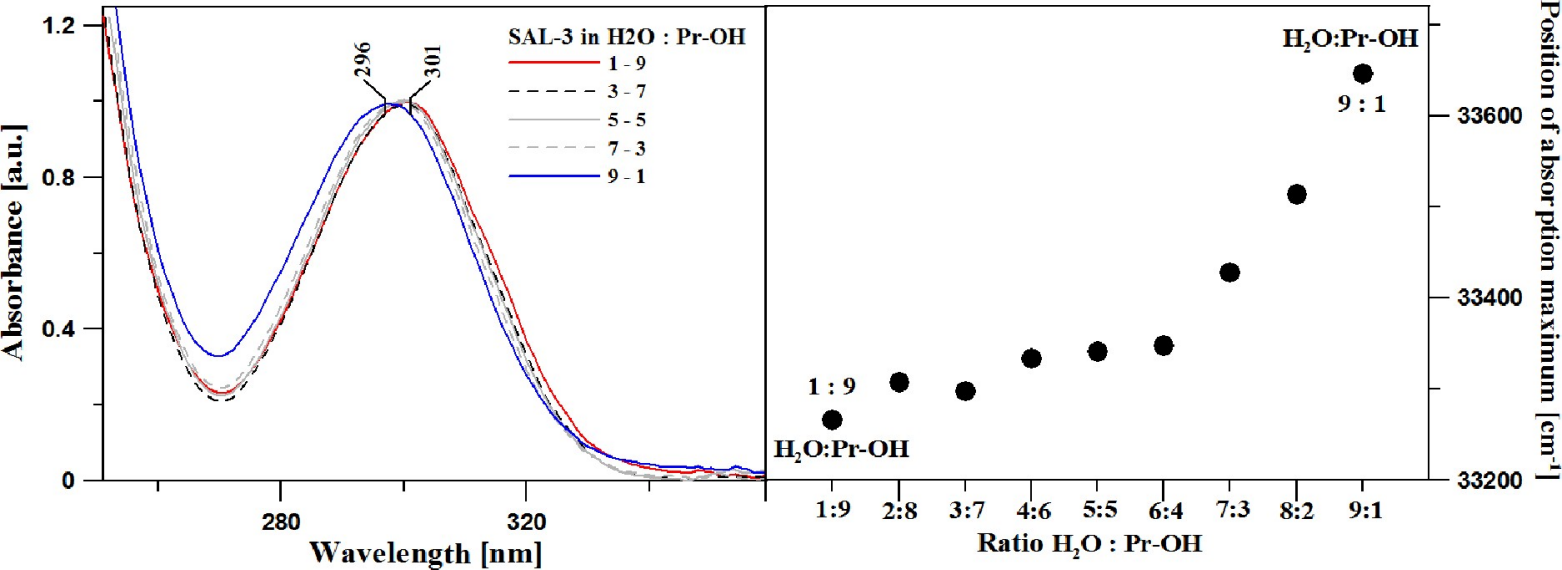
**Panel A:** Electronic absorption spectra for selected ratios of water to propan-2-ol, normalized at the wavelength maximum. **Panel B:** Position of the maximum of the electronic absorption band for the spectra presented in Panel A.

Next, in order to determine the impact of changing polarity/polarizability of the medium on the observed fluorescence effects (presented in Fig 3), Panel A in Fig 6 presents the results of the electronic absorption spectra measurements conducted on SAL-3 in various ratios of water to propan-2-ol.

The spectra were normalized at the absorption band maximum. As we can see, the band maximum, corresponding to the π→π* electronic transition [47] (approx. 300 nm), clearly shifts towards the shorter wavelengths, from 301 to 296 nm, depending on the addition of the polar (protic) solvent. Furthermore, on the longwave side of the bands, at approx. 320 nm, there is a visible slight broadening of the absorption band (affecting its half width) evidencing the coexistence of various forms of the compound (ionic, non-ionic or aggregation effects, although in this case most likely the fact of the presence of aforementioned forms–as will also be discussed further in the text). In turn, Panel B in Fig 6 presents the shift of the absorption maximum (from Panel A) relative to the ratio of the solvents used (water/propan-2-ol). As can be observed, the relationship remains at roughly the same level up to 6:4 (Pr-OH: H_2_O). Above that value, there is a fast shift of the band towards the longwave side and the effect of dual fluorescence is no longer observed in the fluorescence emission spectra (further in the text).

**Fig 7:**
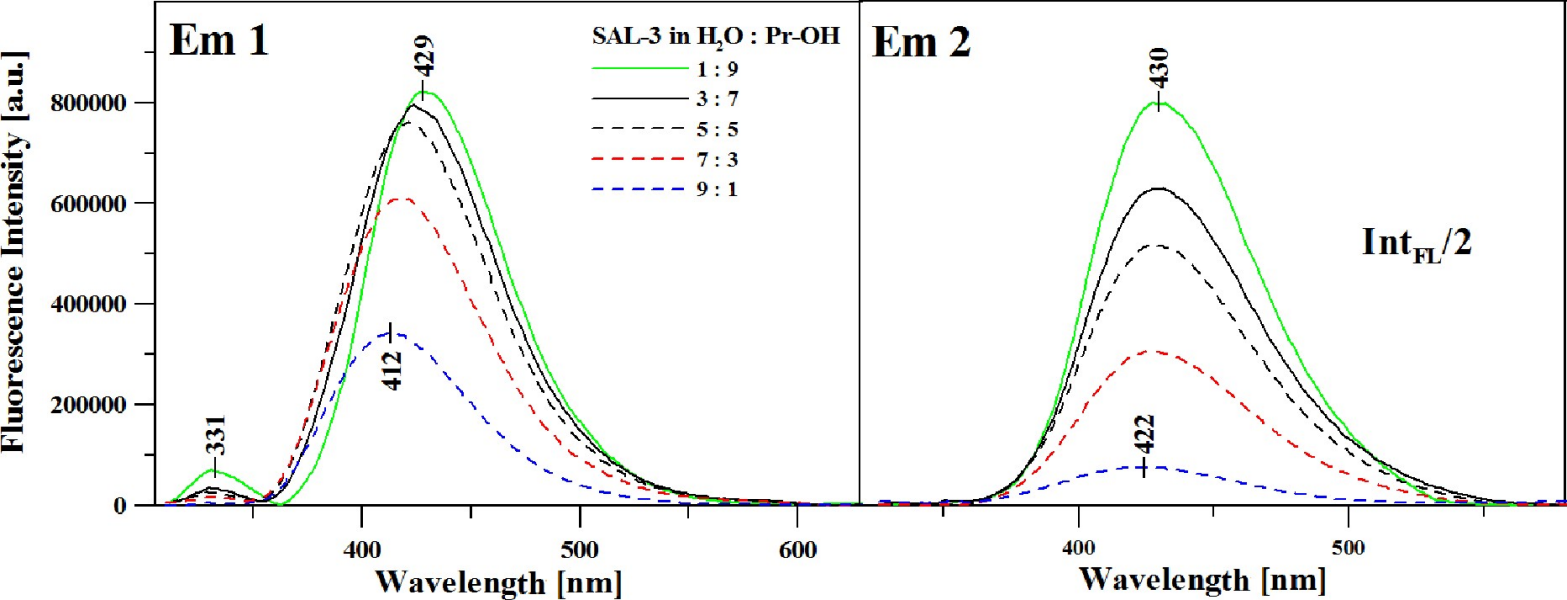
**Panel A:** Fluorescence emission spectra corresponding to the spectra in Fig 6A. The excitation wavelength corresponded to the maximum of the absorption band. **Panel B:** Fluorescence emission spectra analogous to those in Panel A but with the excitation wavelength corresponding to the slopes of the absorption band–as described in the paper.

However, the most noteworthy effects are those presented in Fig 7 (Panel A).

Panels A and B illustrate the fluorescence emission spectra for the studied analogue in various ratios of water to propan-2-ol. Panel A shows the fluorescence emission spectra after excitation at the main maximum of the absorption spectra (as presented in Fig 6, Panel A), i.e. at approx. 300 nm. In this case, we can very clearly observe a very interesting effect of dual fluorescence with the maxima at approx. 331 nm and 429 nm in samples where the solutions contained more propan-2-ol (i.e. more polar and protic media). By comparing the spectra in Fig 7, Panel B (with Fig 6), we can clearly observe that the effect of dual fluorescence in SAL-3 emission spectra occurs up to the H_2_O to Pr-OH ratio of approx. 6:4. The addition of the polar protic solvent into the analyzed system enforced the emergence of dual emission related, in this particular case, to the balance between ionic and non-ionic forms of the analyzed analogue. With decreasing content of propan-2-ol, the band on the shortwave side of the emission spectra quickly disappears, we also observe decreasing intensity of the band with the maximum at approx. 430 nm which also shifts towards shorter wavelengths to approx. 412 nm.

The correlations presented in the graphs used for the calculation of ground and excited-state dipole moments as well as their respective ratios clearly evidence the differentiation between polar protic solvents (in whose presence the effects of dual fluorescence are observed) and non-polar or polar aprotic solvents, in which only a single emission band in the fluorescence spectrum is observed (further in the text). The results obtained in terms of changes in the dipole moments of SAL-3 molecules, as well as the quantum-mechanical [TD]DFT calculations, indicate the fact that the dual emission effects observed for this molecule are caused by the ESIPT effect combined, in this case, with the coexistence of the ionic and non-ionic forms of the analyzed analogue. Panel B in Fig 7 presents the analogical spectra in Panel A, but in this case for at approx. 320 nm (band widening in the absorption spectra corresponding to the ionized form of the given molecule). As can be seen, in this case we no longer observe the effect of dual fluorescence, which excludes the contribution of aggregation effects to the phenomenon–as already discussed above. Or, more precisely, aggregation effects can be said to quench the observed effect of dual fluorescence. These results are consistent with those presented in Figs 1 and 3.

**Fig 8:**
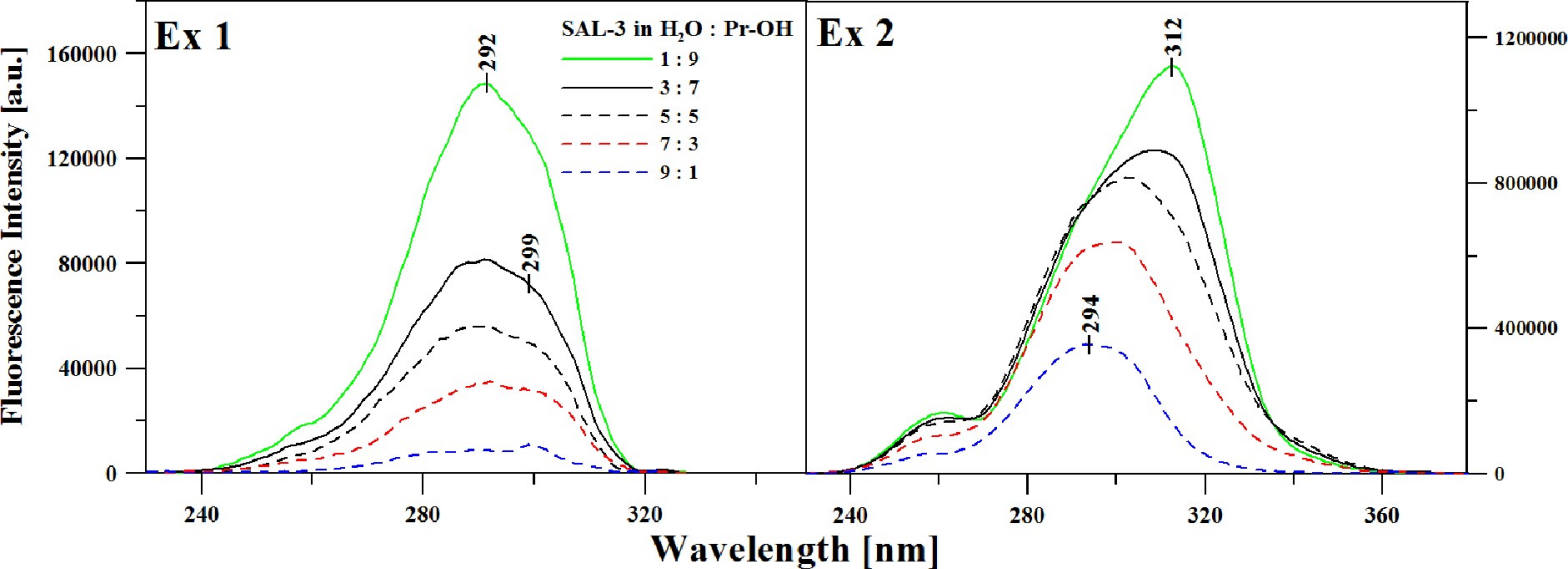
Fluorescence excitation spectra corresponding exactly to the emission spectra in Fig 7. **Panel A:** Excitation spectrum with emission detected at 331 nm; **Panel B:** Excitation spectrum with emission detected at 429 nm.

Fig 8 illustrates the fluorescence excitation spectra corresponding to the emission spectra presented in Fig 7.

In this case, the excitation wavelength corresponded to the first (Panel A) or second (Panel B) maximum in the fluorescence emission spectrum (for dual emission). Fluorescence excitation spectra, due to their selectiveness, display various spectral forms originating from different forms, in this case from ionized or non-ionized forms. As can be observed in Fig 8, depending on the ratio of the solvents used (water:propan-2-ol) and clearly depending on the excitation, we observe the presence of bands which can be associated with either the appropriate type of ionic or non-ionic forms. At the excitation wavelength corresponding to the shortwave fluorescence emission maximum from Fig 7, we observe basically only a change in the intensity of the bands relative to the changing ratio of the solvents. However, in the case of longwave excitation (most likely corresponding to the increasing prevalence of the ionic form), as we add water, we observe that the band is shifted towards longer wavelengths, up to approx. 312 nm, as well as a significant increase in band intensity. The effects correspond to the changes in the absorption/emission spectra and confirm the contribution of various ionic forms of the compound to the fluorescence emission.

Similarly to the aqueous solutions, also in this case the spectra of resonance light scattering (RLS) were measured. Fig 4B presents RLS spectra corresponding to the results in Figs 6–8 for the analyzed SAL-3 solutions in various solvents. As shown, with increasing content of water we observed a significant increase in the intensity of the RLS spectra, which may also confirm the presence of aggregation effects that may coincide with various forms of the compound, e.g. ionic and non-ionic. As can be seen in Fig 4B, the RLS spectra for the respective ranges of the ratio vary in terms of intensity and shape. One can also note the oscillative structure of the observed RLS spectra indicating the potential presence of different ionic and non-ionic structures depending on the changes in the quantitative proportions of the solvents. It is also noteworthy that for the solvent ratio of 5:5 and with increasing amount of water, there is a band between 300–350 nm which very quickly gains in intensity. It can be associated (as confirmed by the above results) with the growing content of ionic forms of SAL-3 in the analyzed system. The remaining RLS spectra (in the longwave direction) change in intensity but not in shape, which further confirms that the analyzed system may contain both mentioned forms of the compound.

### 3.5 TCSPC study

Time-resolved spectroscopic measurements were performed for SAL-3 in the same pH range as in the experiments described above. The excitation wavelength used was 294 nm as it is the most effectively absorbed by the most intensive absorption component (the most intensive peak with the maximum at approx. 300 nm–presented in Fig 1 or 6A). It will also be absorbed by the other two components–however, significantly less efficiently. Fluorescence emission was measured for wavelengths higher than 408 nm, i.e. only one fluorescence component was monitored–the one characterized by the greater wavelength.

**Fig 9:**
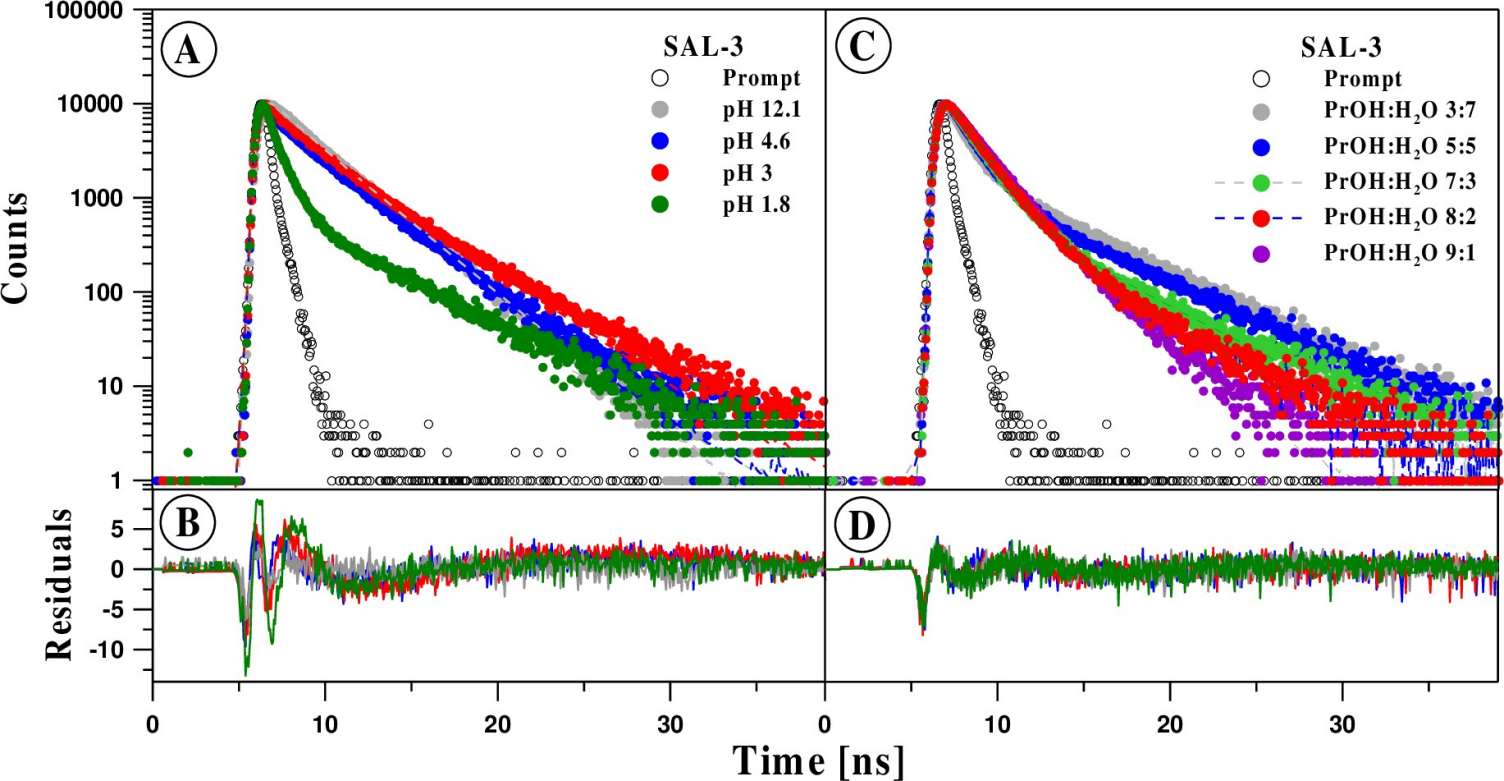
**Panels A-B:** TCSPC analysis for SAL3 in water at various pH. The top panel presents the decay of fluorescence intensity. Black color denotes the apparatus profile. Points correspond to measurement results and the continuous line to the double component analysis. The bottom panel presents the distribution of residuals. The excitation wavelength was 294 nm. **Panels C-D:** TCSPC analysis for SAL-3 in water in various solution compositions. The top panel presents the decay of fluorescence intensity for a selected propanol content. Black color denotes the apparatus profile. Points correspond to measurement results and the continuous line to the double component analysis. The bottom panel presents the distribution of residuals. The excitation wavelength was 294 nm.

The analysis of fluorescence intensity decay was conducted using deconvolution for a 1, 2, and 3 component model. For all the analyzed pH values, the two-component model proved to be optimum. The analysis based on a singular decay was insufficient, whereas the addition of the third component did not significantly improve the quality of fit (Fig 9).

**Fig 10:**
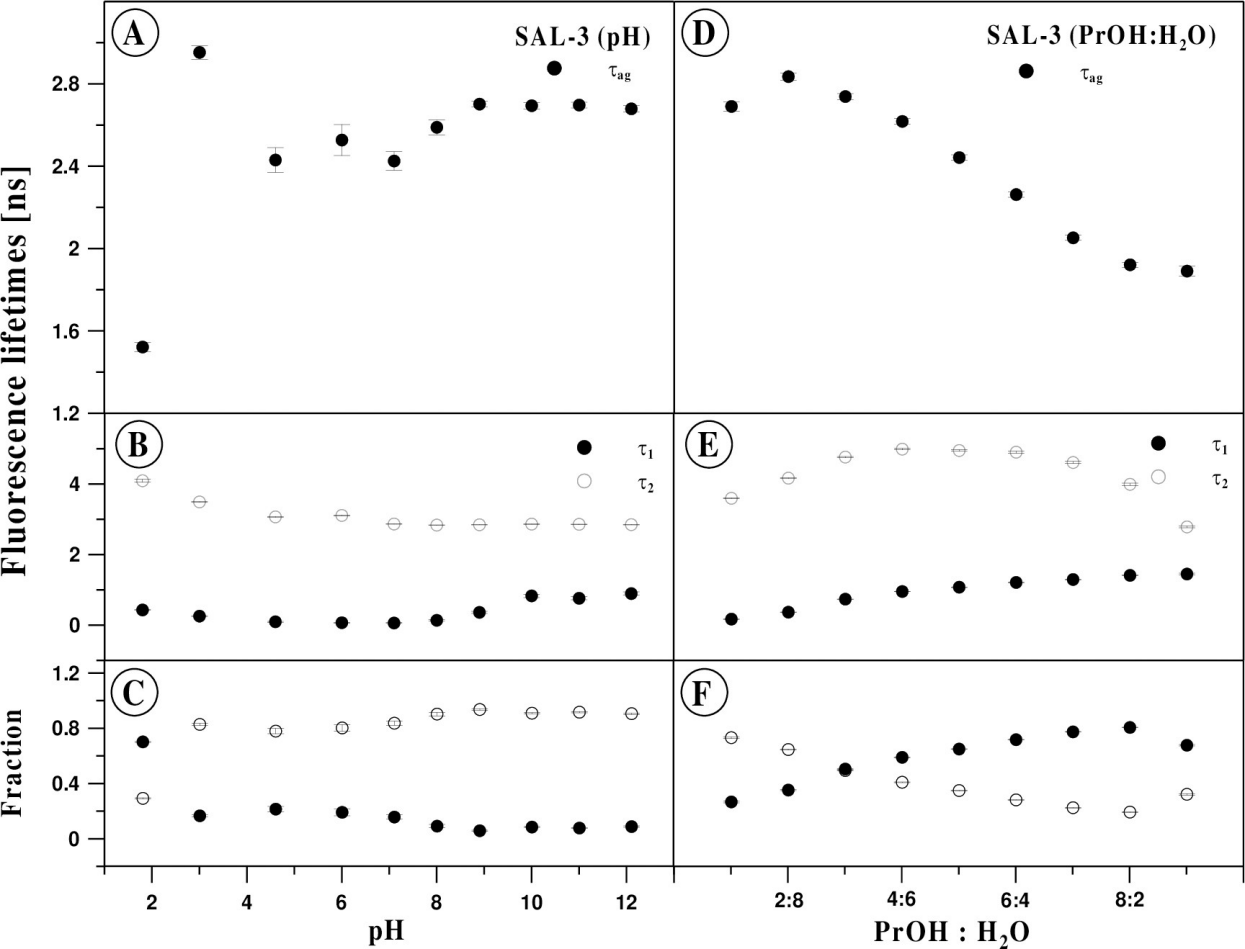
**Panels A-C:** Effect of pH on the fluorescence lifetime for SAL3 in water. Panel A presents fluorescence lifetimes of the mean values observed at various pH levels, while panel B shows fluorescence lifetimes of the first (τ1) and the second (τ2) component, observed for various pH. The dependences of the fraction of both components in pH are presented in Panel C. The data analysis was carried out for the two-component model (Eq (1) for i = 2) for data presented in Fig 9A and 9B. **Panels: D-F:** Effect of the content of propan-2-ol on SAL-3 fluorescence lifetime. Panel A presents fluorescence lifetimes of the mean values observed in various propanol mixtures, while panel B shows fluorescence lifetimes of the first (τ1) and the second (τ2) component. The dependences of the fraction of both components in solution composition are presented in Panel C. The data analysis was carried out for the two component model (Eq (1) for i = 2) for data presented in Fig 9C and 9D.

In all the analyzed cases, the long-life component had a characteristic time of approx. 3 ns, which decreased slightly with growing pH. For the second, short-life component, considerably greater pH-dependent variability was observed. At low pH, it was approx. 0.1 ns, whereas at high pH it increased to approx. 0.9 ns. A clear change in the lifetime of this component was observed for pH in the range of 8–10 (Fig 10, Panel B).

The contribution of the long-life component was greater than that of the short-life component, approx. 80%, and increased to approx. 90% with growing pH. Only for the lowest pH levels was an inversely proportional relationship between the components observed, as in this case the contribution of the long-life component was only approx. 30%. A significant change of this parameter was also observed at around pH 7 (Fig 9, Panel C). The described values have an obvious impact on the mean fluorescence lifetime which increased from approx. 1.5 ns to approx. 2.7 ns with increasing pH. In this case, the most significant change was observed for pH 2 and pH 7 (Panel A in Fig 10, Table 2).

**Table 2:** Fluorescence lifetimes of SAL-3 in different aqueous solvents at different pH with F408 cut-off filter and diode 294 nm.

SAL-3 in different pH		
F408		
pH	α	τ ± Δτ
1.8		1.523 ± 0.023
3		2.955 ± 0.033
4.6		2.432 ± 0.060
6		2.529 ± 0.075
7.1		2.427 ± 0.045
8		2.591 ± 0.036
8.9		2.703 ± 0.014
10		2.696 ± 0.016
11		2.699 ± 0.014
12.1		2.681 ± 0.016

An identical measurement system was used to determine the impact of the solvent’s (polarity/polarizability) on the spectroscopic parameters of SAL-3 (Fig 9D–9F). In particular, the excitation wavelength and range of emission wavelengths considered were the same as in the time-resolved experiments discussed above. The solvent was a mixture of water and propan-2-ol constituting between 10% and 90% of the solvent’s volume. Similarly, to the previous cases, the analysis was conducted based on deconvolution in a 1, 2, and 3 component model of fluorescence intensity decay. In each of the cases considered, the two-component model proved the most adequate, which evidences the presence of two classes of fluorophores. The contribution of the first, characterized by a shorter fluorescence lifetime, was 27% for the low– 10% content of propanol, and increased to 81% at the 80% concentration. A further increase of the concentration resulted in a decrease of the component’s contribution–to 68% (Fig 9, Panel F). The fluorescence lifetime of this component increased monotonically with the increasing concentration of propan-2-ol in the entire range of the concentrations considered, form 0.18 ns to 1.5 ns. The lifetime of the second constituent, characterized by a linger fluorescence lifetime, was 3.6 ns in 10% propanol, and increased to 5 ns in the concentration range of 40–60%, after which it decreased again to 2.8 ns as the propanol concentration continued to increase (Fig 10, Panel D). The mean fluorescence lifetime, determined on the basis of said results from Eq 3, showed a monotonic decrease with the growing concentration of propanol, from 2.8 to 1.9 ns within the concentration range of 20–90%. Decreasing the concentration of this component to 10% resulted in a slight decrease in the mean fluorescence lifetime to 2.7 ns (Fig 10, Panel C, Table 3).

**Table 3:** Fluorescence lifetimes of SAL-3 in different aqueous solvents at different pH with F408 cut-off filter and diode 294 nm.

SAL-3 in H_2_O : Pr-OH		
F408		
pH	α	τ ± Δτ
9 : 1		2.693 ± 0.023
8 : 2		2.837 ± 0.016
7 : 3		2.741 ± 0.014
6 : 4		2.620 ± 0.014
5 : 5		2.444 ± 0.014
4 : 6		2.264 ± 0.013
3 : 7		2.054 ± 0.012
2 : 8		1.923 ± 0.013
1 : 9		1.893 ± 0.023

The above results confirm the possible e existence/coexistence of two forms of the analyzed compound, ionic and non-ionic, depending on the changes in the pH of the system or the polarity of the medium used.

### 3.6 Computational results

**Fig 11:**
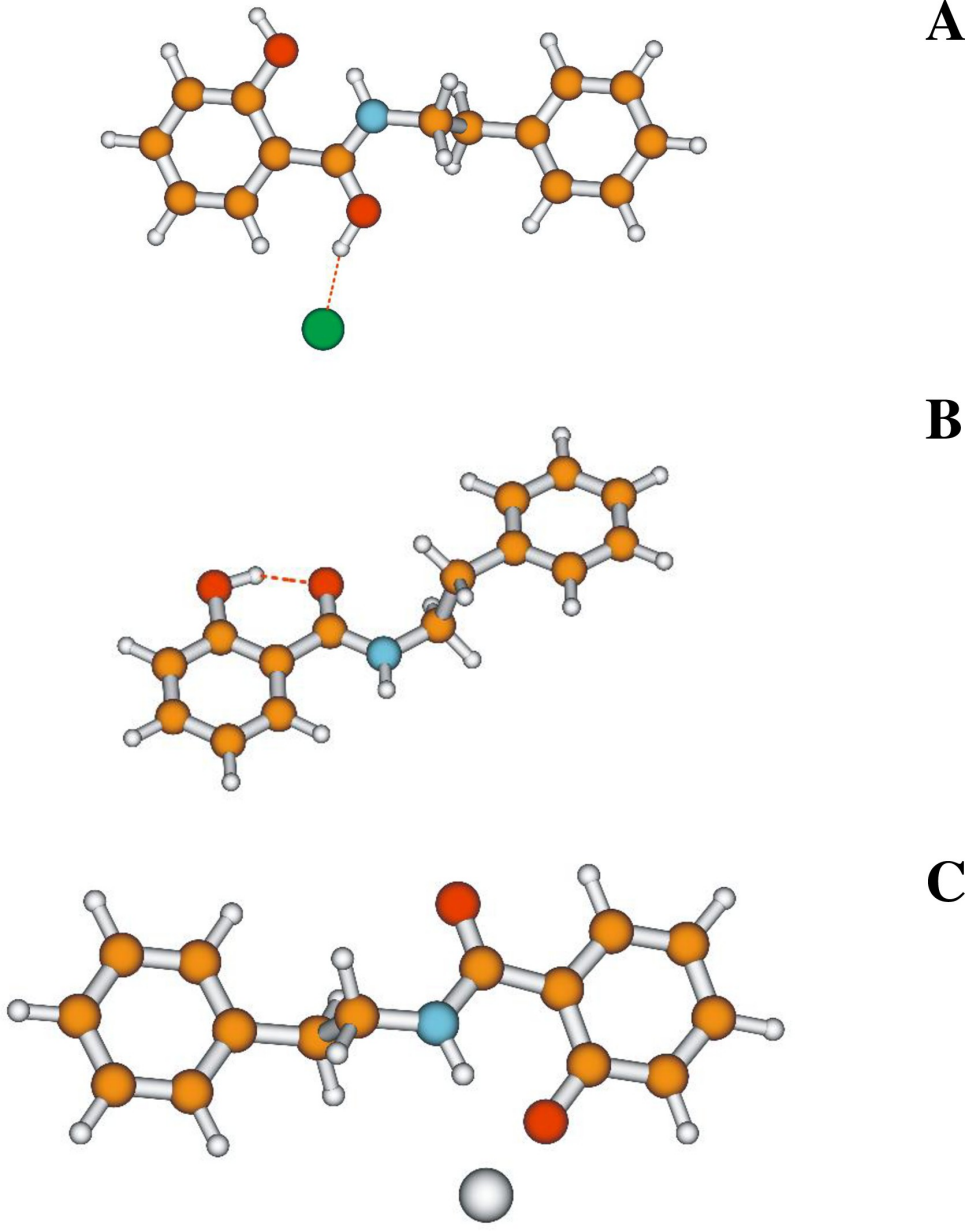
Ground state structures of various protonation forms of the SAL-3 molecule as optimized using B3LYP/aug-cc-pVDZ and PCM treatment with water as a solvent. Hydrogen bonds depicted by red dashed lines. **Panel A:** SAL-3H^+^ form, **Panel B:** SAL-3 form and **Panel C:** SAL-3^-^ form.

The conducted quantum-mechanical calculations corroborated the conclusions reached on the basis of the experimental data. In the case of SAL-3, we are dealing with a pH-dependent balance/equilibrium of various forms of the compound. At very low pH, the SAL3H^+^ form is predominant (Fig 11A), whereas at high pH, the SAL3^-^ form is prevalent (Fig 11C).

At the remaining pH values, the undissociated form SAL-3 is predominant. The locations and intensities obtained from the [TD]DFT calculations were highly consistent with the changes observed in the absorption and fluorescence spectra. The observed shift of the absorption maximum towards longer wavelengths with increasing pH, the shift of fluorescence emission bands towards longer wavelengths accompanied by a decrease in intensity between medium and very low pH levels, as well as the shift towards shorter wavelengths between medium and high pH values–were all confirmed in the results of [TD]DFT calculations (Fig 11 and Table 4).

**Table 4:** TD-DFT predictions for various protonation forms of the SAL-3 molecule in a solvent (water) treated as per the PCM approach. Energetics given in a nm scale, dimensionless oscillatory strengths given in parentheses. Low-lying absorbing states shown as well as the lowest excited state for emission.

Form	Vertical absorption	Vertical emission
**SAL-3H**^**+**^	303 (0.15); 297 (0.05)	443 (0.08)
**SAL-3**	293 (0.18)	423 (0.27)
**SAL-3**^**-**^	318 (0.17)	376 (0.24)

### 3.7 Estimation of ground state and excited state dipole moment

In order to determine how the dipole moments of the analyzed molecule vary between its ground state and excited state, in the present study, the ground and excited-state dipole moment of the SAL-3 molecule was estimated for two different positions of the fluorescence maxima (Em 1 –maximum at approx. 330 nm, Em 2 –maximum at approx. 430 nm). In fluorescence measurements for polar solvents, the analyzed molecule produced two bands of varying intensity, whereas in nonpolar solvents, we observed only a single band, as already discussed above in this text. The positions of the SAL-3 molecule’s absorbance and fluorescence maxima in various solvents are presented in Table 5.

**Table 5:** Positions of the absorption spectra and fluorescence maxima for SAL-3 in different solvents.

Solvent	Maximum absorbance [nm]	I Maximum fluorescence [nm]	II Maximum fluorescence [nm]	I Stokes shift [cm^-1^]	II Stokes shift [cm^-1^]
H_2_O	330	370	430	3276.003	7047.216
Propan-2-ol	301	330	430	2919.561	9966.777
Ethanol	302	332	432	2992.101	9964.435
DMSO	303	332	433	2882.818	9908.612
DMF	303	335	432	3152.554	9855.152
Butan-1-ol	302	333	432	3082.553	9964.435
Acetonitrile	304	333	436	2864.707	9958.957
Methanol	302	332	431	2992.101	9910.727
n-Heptane	308		436		9531.753
n-Hexane	309		436		9426.68
Chloroform	306		434		9638.264

The ground and excited dipole moment for SAL-3 was estimated using two methods with the use of two solvent polarity functions F_1_(ε, n) and F_2_(ε, n) (Eqs 6 and 7) as well as the microscopic solvent function $E_{T}^{N}$ (Eq 13) for various solvents (n-heptane, n-hexane, chloroform, butan-1-ol, propan-2-ol, ethanol, DMF, DMSO, methanol, acetonitrile, H_2_O). The values of the light refraction index, the dielectric constant ε, and the functions of F_1_(ε, n), F_2_(ε, n) and $E_{T}^{N}$ are presented in Table 6.

**Table 6:** Solvent parameters F_1_(ε,n), F_2_(ε,n), dielectric constant ε, index of refraction n and $E_{T}^{N}$ for different solvents.

Solvent	ε	n	F_1_(ε,n)	F_2_(ε,n)	$E_{T}^{N}$
H_2_O	80.10	1.3333	0.914	0.532	1.000
Propan-2-ol	20.18	1.3772	0.781	0.476	0.552
Ethanol	25.30	1.3594	0.817	0.49	0.654
DMSO	47.24	1.4773	0.842	0.529	0.444
DMF	36.70	1.4305	0.836	0.515	0.386
Butan-1-ol	17.80	1.3993	0.753	0.467	0.586
Acetonitrile	36.64	1.3416	0.862	0.508	0.460
Methanol	33.00	1.3265	0.856	0.502	0.765
n-Heptane	1.92	1.3876	-0.001	0.087	0.012
n-Hexane	1.89	1.3723	0.002	0.085	0.006
Chloroform	4.81	1.4429	0.372	0.286	0.259

**Fig 12:**
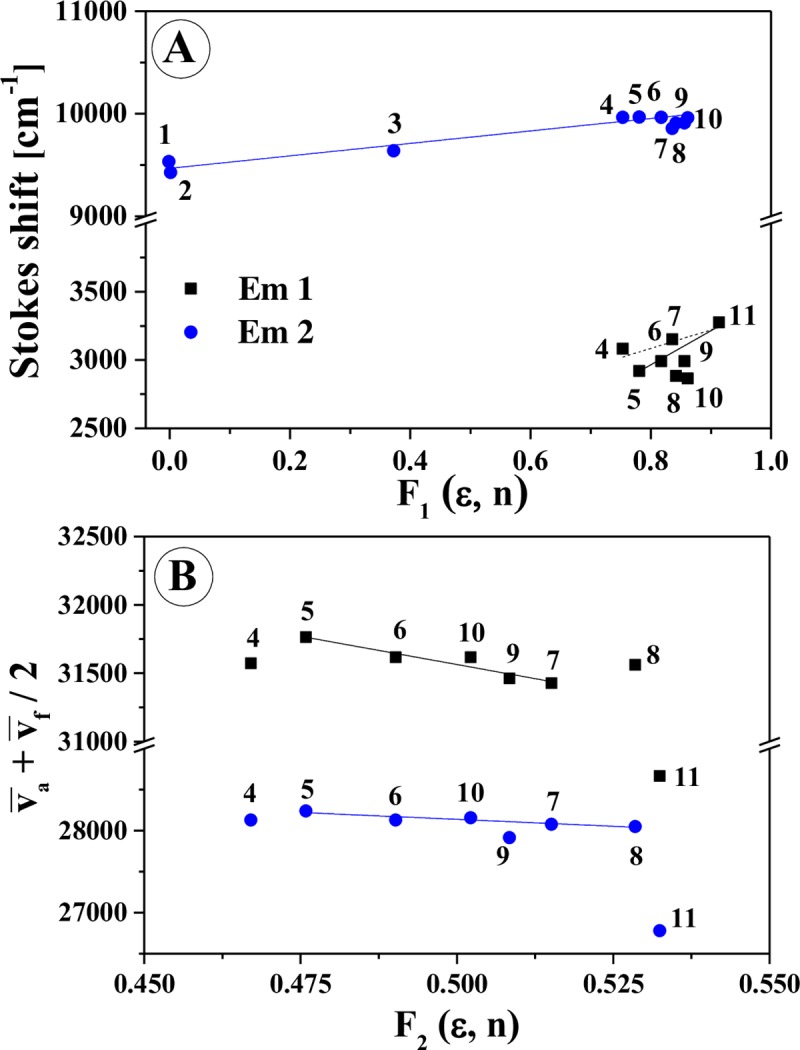
Stokes shift to F_1_(ε,n) using Bakhshiev’s equation (**panel A**), $({\bar{v}}_{a}+{\bar{v}}_{f})/2$ to F_2_(ε,n) using Kawski-Chamma-Viallet’s equation (**panel B**) for SAL-3 dissolved in different solvents (1—n-heptane, 2—n-hexane, 3 –chloroform, 4—butan-1-ol, 5—propan-2-ol, 6—ethanol, 7—DMF, 8—DMSO, 9—methanol, 10 –acetonitrile, 11—H_2_O). Black square–**Em 1** (maximum of fluorescence ~ 330 nm), blue circle–**Em 2** (maximum of fluorescence ~ 430 nm).

**Fig 13:**
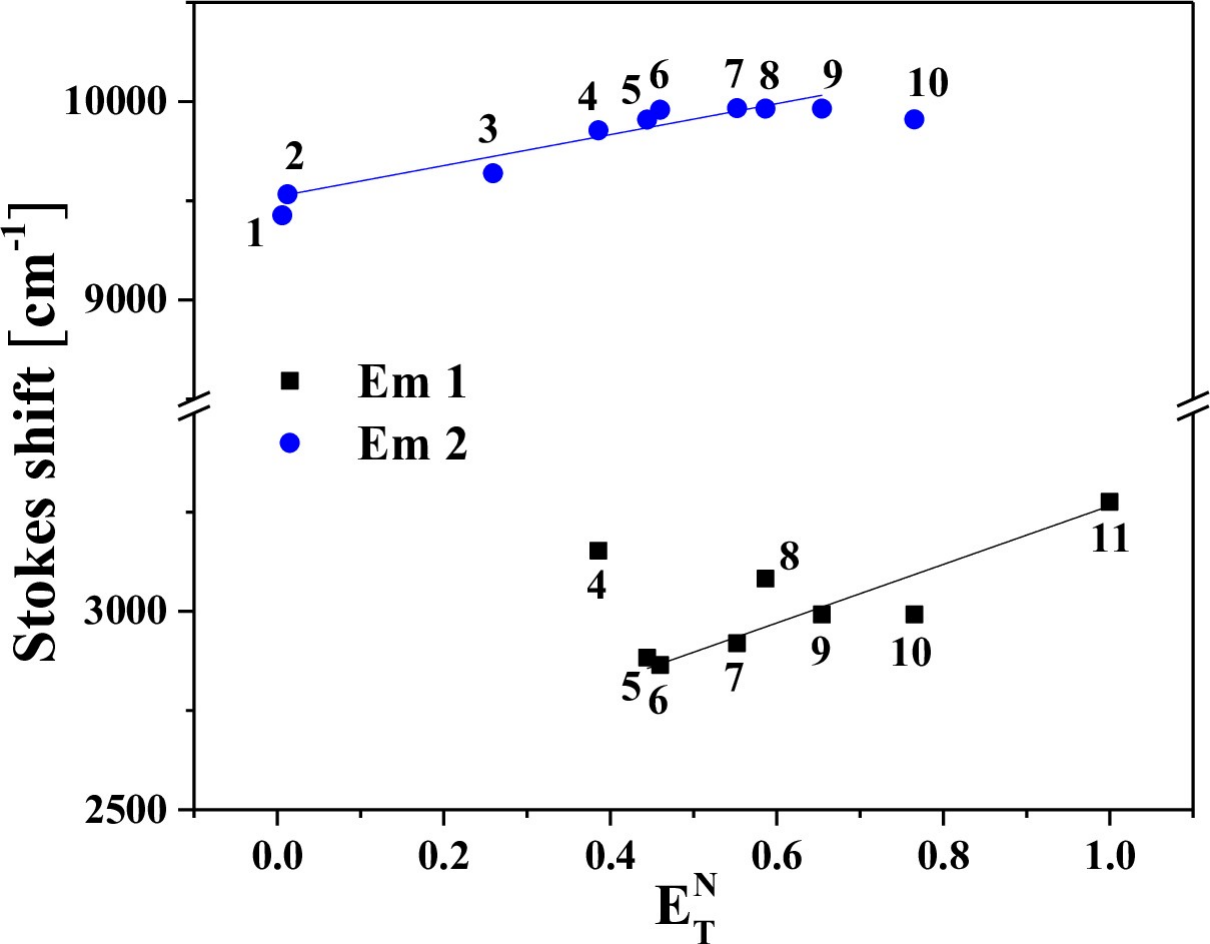
Stokes shift variation with the normalized value of solvent polarity $E_{T}^{N}$ for SAL-3. Black square–I Stokes Shift, red circle–II stokes shift in different solvents (1—n-hexane, 2—n-heptane, 3 –chloroform, 4—DMF, 5—DMSO, 6—acetonitrile, 7 –propan-2-ol, 8 –butan-1-ol, 9—ethanol, 10 –methanol, 11—H_2_O).

The graph presenting the Stokes shifts for the F_1_(ε, n) function and $({\bar{v}}_{a}+{\bar{v}}_{f})/2$ for the F_2_(ε, n) function are presented in Fig 12 (Panels A and B), whereas the correlation between the Stokes shift and the $E_{T}^{N}$ parameter is presented in Fig 13.

In method 1, based on the publications by Bakshiev and Kawski-Chamma-Viallet, in the case of the first fluorescence maximum (Em 1) the estimated value of Δμ for polar solvents was 4.493 and 3.314 depending on the linear fit applied (Table 7).

**Table 7:** Onsager cavity radius a_0_ for SAL3. Slopes S_1_ and S_2_ determined by Stokes shift and $({\bar{v}}_{a}+{\bar{v}}_{f})/2$ in the function of F_1_(ε,n) and F_2_(ε,n), ground state μ_g_, excited state μ_e_, change in dipole moment Δμ and coefficient of determination r^2^ for SAL-3.

	a_0_ [Å]	m_1_	m_2_	μ_e_/μ_g_	μ_e_ [D]^a^	μ_g_ [D]^b^	Δμ [D]^c^	r^2^
**Em 1**	4,336	2492.268	-8268.586	1.863	9.700	5,207	4.493	0.72 : 0.90
		1355.502	-8268.586	1.392	11.763	8.450	3.314	0.56 : 0.90
**Em 2**		608.444	-3367.164	1.441	7.253	5.033	2.220	0.96 : 0.87

1 D = 3.33564 · 10^−30^ C·m = 10^−18^ statC · cm

^a^ Value calculated from the plot of Stokes shift vs. F_1_(ε,n)

^b^ Value calculated from the plot of $({\bar{v}}_{a}+{\bar{v}}_{f})/2$ shift vs. F_2_(ε,n)

^c^ The dipole change calculated from the experimental values of μ_g_ and μ_e_

Whereas in the case of the second fluorescence maximum (Em 2), the change in the dipole moment was Δμ 2.220. Table 6 also presents the values of slopes (m_1_ and m_2_) and coefficients of determination r^2^. In the case of the graph in Panel B, good fit of the model was observed in both cases (r^2^ in the range of 0.87–0.90), whereas in the case of the Panel A graph, a somewhat worse fit was observed for the first fluorescence maximum (Em1) (r^2^ = 0.56).

In the second method based on the Dimroth-Reichardt methodology, the changes of the dipole moment values for the two analyzed shifts were similar: for Em 1 Δμ was 1.344, for Em 2 Δμ was 1.384 (Table 8).

**Table 8:** Slope m, change in dipole moment Δμ and coefficient of determination r^2^ for SAL-3.

	M	Δμ [D]	r^2^
**Em 1**	737.859	1.344	0.98
**Em 2**	780.620	1.383	0.89

The value of r^2^ was within the range of 0.89 to 0.98, which evidences good linear fit. As could be expected, the value of the excited-state dipole moment was higher than the ground-state dipole moment, which indicates that the SAL-3 molecule is more polar in its singlet excited state as compared to its ground state. The above calculations of the changes in the SAL-3 molecule dipole moments suggest that solvatochromic shifts lead to an important effect of crisscrossing states with different distributions of electronic density relative to the polarity of the medium used. In polar solvents, electronic states with high dipole moments (often the states associated with e.g. excited-state proton transfer) are stabilized relative to the low dipole moment states. If the energies of such states are similar, a change in the polarity/polarizability of the medium may trigger a reversal of the states’ order. Photochemical properties are determined by the character of the lowest excited state with the given multiplicity (Kasha’s rule), consequently they can be affected (as observed in our study) by very significant changes of solvent polarity. And in the case of our study, we observed effects associated with the phenomenon of dual fluorescence in SAL-3 molecules with the changing medium polarity.

It can be therefore preliminarily posited that the presence of the interesting effect of dual fluorescence observed for the SAL-3 compound, based on the results presented below in Figs 1–13 relative to both stationary spectroscopy and fluorescence lifetime measurements as well as, most importantly, the conformational potential of the SAL-3 molecule, is connected to the effect of excited-state intramolecular proton transfer (ESIPT) in this particular particle. Let us observe that for SAL-3, the effects of dual fluorescence occur in a medium (protic) where with adequate rotation of the molecule there is a possibility of forming a hydrogen bond. In environments characterized by the prevalence of ionized forms of SAL-3, i.e. e.g. at pH higher than 6.5 or media composed of mixed solvents with increasing water content, the effect tends to disappear very quickly. Both the techniques of resonance light scattering (RLS) and band decomposition with the analysis of the contribution of the respective components also excluded, with high probability, the contribution of chromophoric aggregation to the induction of the discussed effect. In fact, the latter seems to be a quenching factor in this case. As mentioned above, in the emission spectra registered after excitation at the wavelength corresponding to the band which may partially originate from aggregated forms, we observed only a single fluorescence emission band.

The ionized form, prevalent at pH higher than 6.5 (with excitation at the wavelength of the absorption band maximum), corresponds to longwave fluorescence emission. The non-ionized form, present at pH lower than 6.5 (mainly at very low pH levels), corresponds to a single shortwave fluorescence emission band emerging in protic media. In such media, SAL-3 molecules can occur primarily with the ionized–OH group. In protic media (pH under 6.5) as well as in low concentrations, where the prevalent forms of the analyzed system are monomeric (ionic or non-ionic), the aforementioned hydroxyl group is located on the right-hand side of the carbonyl group and an intramolecular hydrogen bond is formed. Along the same, in the excited state, a proton is transferred to the–OH group and a carbonyl group is formed in its place, whereas an–OH group is formed in the original position of the carbonyl group (see Fig 2B and 2C and Fig 11).

## 4. Conclusions

The results presented in this paper obtained primarily with the use of stationary spectroscopy (measurements of absorption and fluorescence spectra), time-resolved spectroscopy (TCSPC measurements of fluorescence lifetimes), as well as resonance light scattering (RLS) measurements, indicate that the fluorescence emission spectra of the SAL-3 compound belonging to the group of salicylic derivatives are characterized by a very interesting effect of dual fluorescence. The discussed effects are present at pH ranges lower than physiological and at low concentrations of the analyzed analogue. At pH higher than physiological, only a single fluorescence emission maximum is observed. At low pH, we observe widening of the main fluorescence emission band on the shortwave side and a significant shift of the main emission band towards longer wavelengths. In the case of mixed solvents, we begin to observe a clear effect of dual fluorescence with increasing content of the protic solvent. In the case of dual emission, the respective bands are present at the wavelengths of approx. 330 nm and 430 nm. The RLS results of synchronic spectra measurements and interpretation of the bands identified in the course of decomposition of selected absorption spectra, also clearly indicate decay of the effect in systems with increasing concentrations of the compound. The measurements of fluorescence lifetimes indicate that for the pH ranges where said fluorescence effects are observed, as well as for adequate proportions of solvents that allow the effect of dual fluorescence, the observed fluorescence lifetimes are only slightly different from the ranges registered in systems where the effects are not present. However, in (mixed solvent) media where dual fluorescence does occur, we observe an additional lifetime component corresponding to the shortwave form of the emission.

Therefore, after analyzing the presented results, we posited that the observed fluorescence phenomena are related to the effects of excited-state electron transfer (ESIPT) taking place in the molecule. The mechanism of dual fluorescence in SAL-3 is primarily due to the coexistence of its ionized and non-ionized forms, which become predominant at pH levels higher/lower than 6.5. The ionized form corresponds to a single longwave band of fluorescence emission. The non-ionized form, having assumed the correct molecular conformation with the hydroxyl group from the hydroxybenzene ring on the side of the carbonyl group, forms a hydrogen bond which facilitates the processes of excited-state proton transfer.

To recapitulate, it should once again be strongly emphasized that the effects of excited-state proton transfer taking place in SAL-3 molecules are of key significance in the context of the observed fluorescence phenomena. As such, they will be further researched in future studies aimed at developing a more exact theoretical model of the phenomena observed in this group of new SA analogues. The ability to provide a concise description of the mechanisms applicable to those particular compounds may contribute to the explanation and better utilization of the mechanisms of their bioactivity. Additionally, doe to their particular photophysical properties, the compounds from this group may also potentially be used as effective fluorescence probes or molecular switches, which continue to be in high demand in the context of molecular biology.

## Supporting information

S1 Fig Topographical and nanomechanical AFM mapping of SAL-3 sample (5 μm scan length). (PDF)

S2 Fig 3D surface topography of the sample from S1 Fig. (PDF)

S3 Fig Topographical and nanomechanical AFM mapping of a SAL-3 sample (2 μm scan length). (PDF)

S4 Fig Topographical and nanomechanical AFM mapping of a SAL-3 sample (2 μm scan length). (PDF)

S5 Fig Topographical and nanomechanical AFM mapping of a SAL-3 sample (1 μm scan length). (PDF)

S6 Fig Example of electronic absorption spectra decomposition for SAL-3 at pH 7.4. Solid black line–experimental spectrum; solid grey line–spectrum fitted with the decomposition components; blue, red, and green lines–respective components of the given band after decomposition.
(PDF)

S7 Fig Graphic symbols. Top–variety of the possible forms of the investigated SAL-3 derivative. Bottom–Fluorescence emission spectra of SAL-3 recorded in H_2_O : 2-propanol mixture with dual emission marked in green.
(PNG)
