# Supplementary figures and images for: Spectroscopic and theoretical studies of fluorescence effects induced by the ESIPT process in a new derivative 2-Hydroxy-N-(2-phenylethyl)benzamide – Study on the effects of pH and medium polarity changes
Source: PLoS One. 2020 Feb 25;15(2):e0229149. doi: 10.1371/journal.pone.0229149 (PMC7041845; doi:10.1371/journal.pone.0229149)

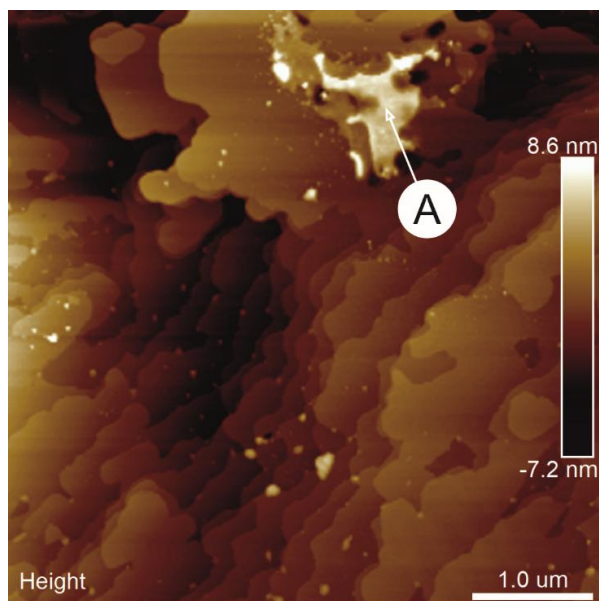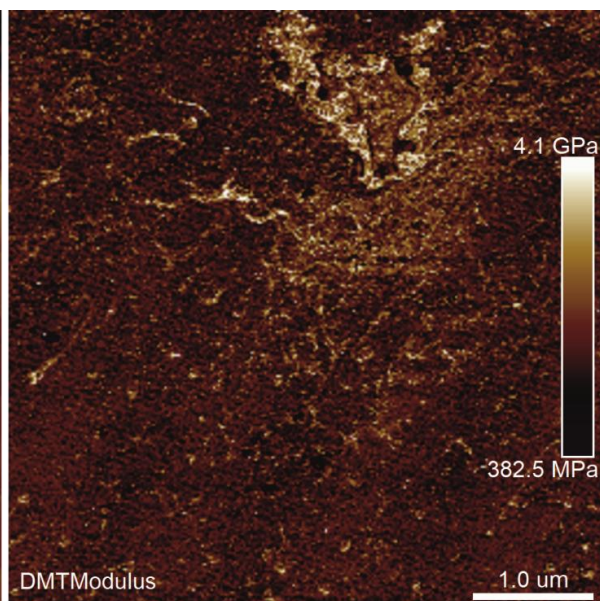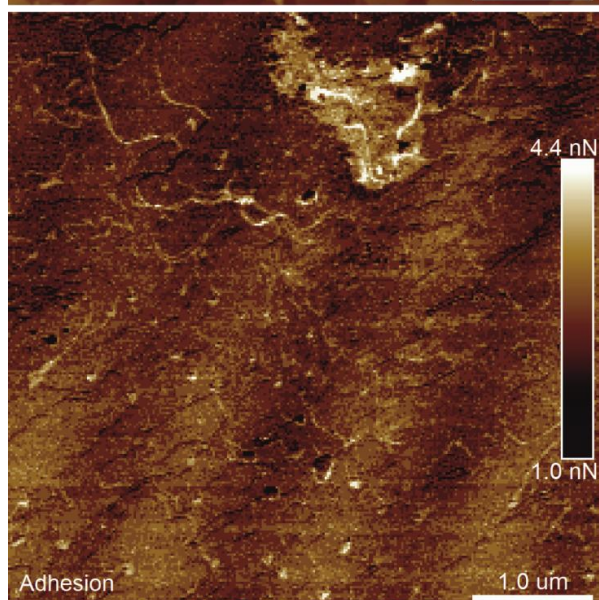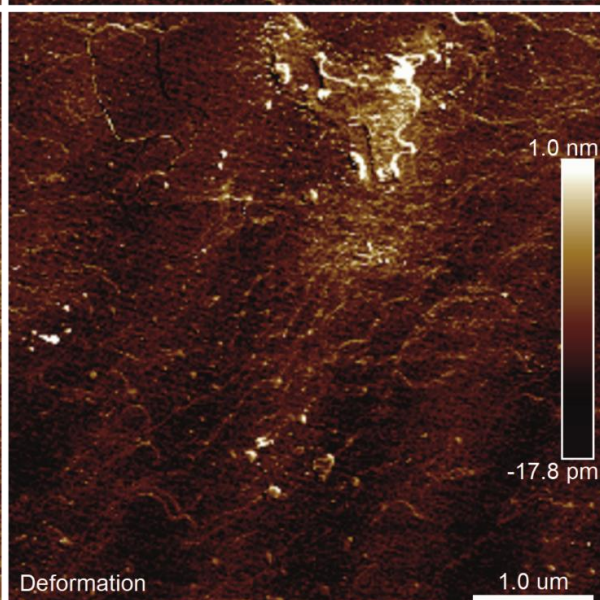

Supplement: S1 Fig — (PDF) [file pone.0229149.s001.pdf]

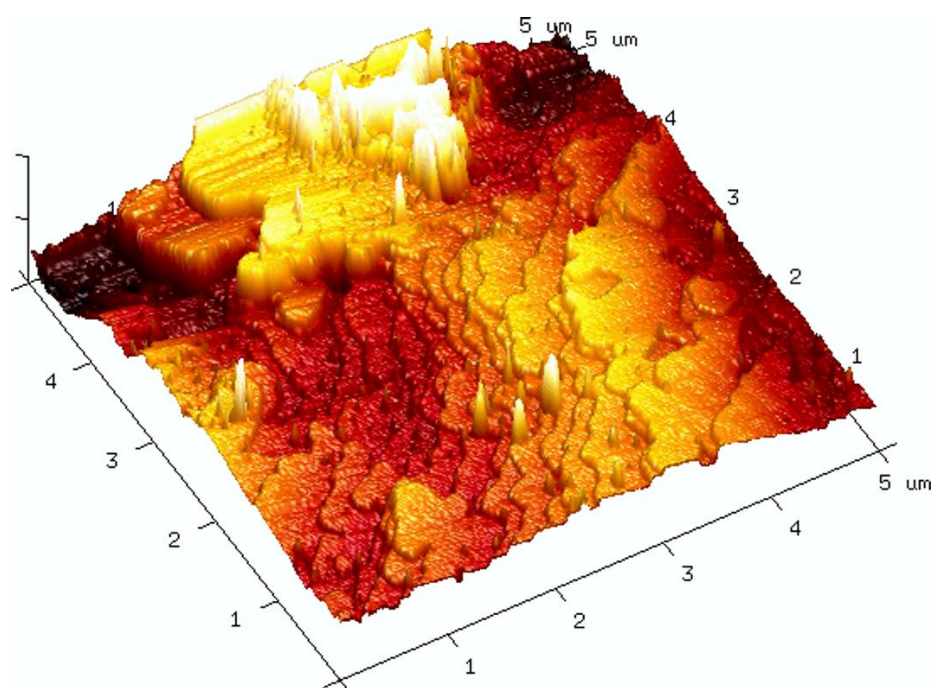

Supplement: S2 Fig — (PDF) [file pone.0229149.s002.pdf]

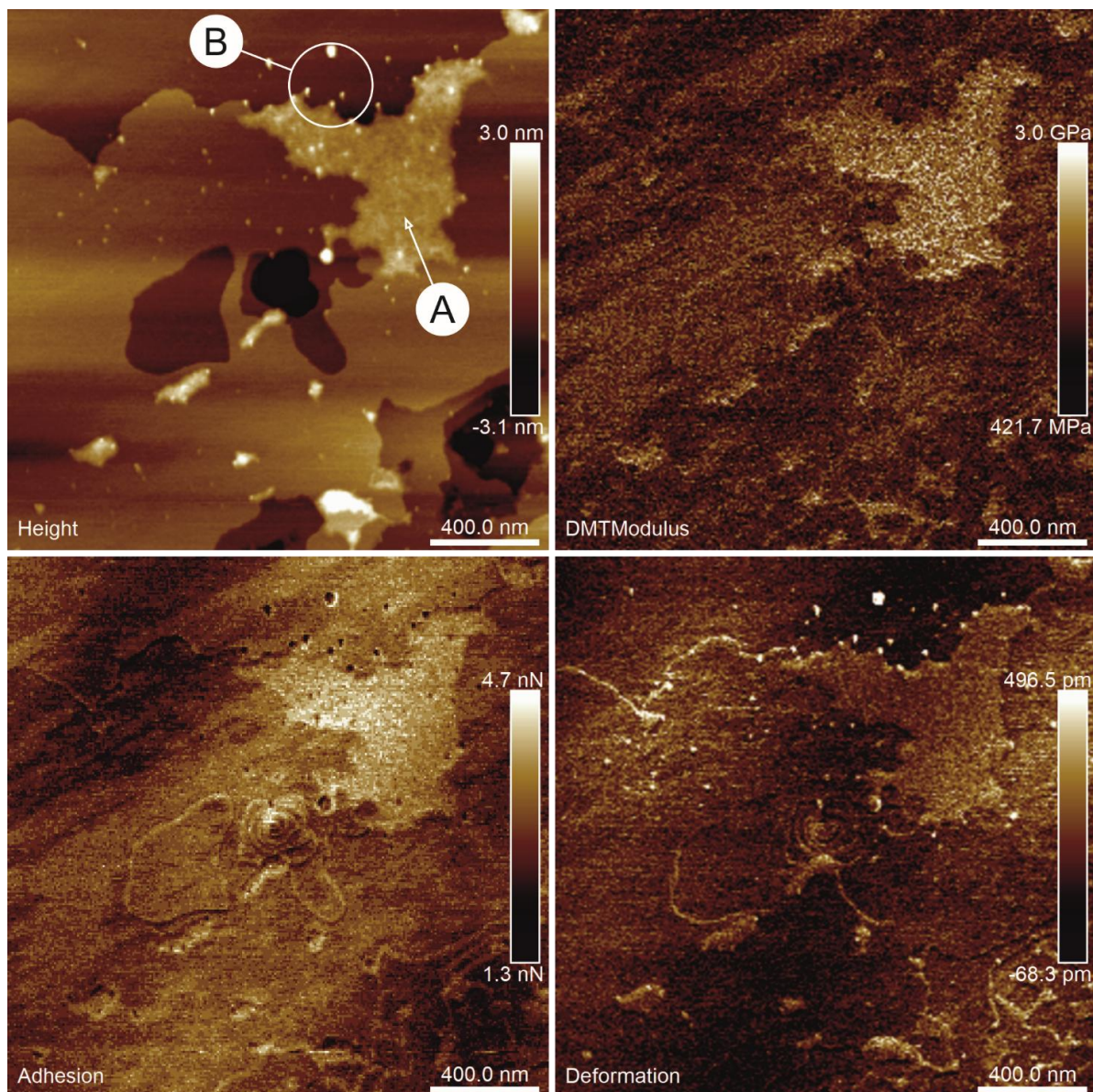

Supplement: S3 Fig — (PDF) [file pone.0229149.s003.pdf]

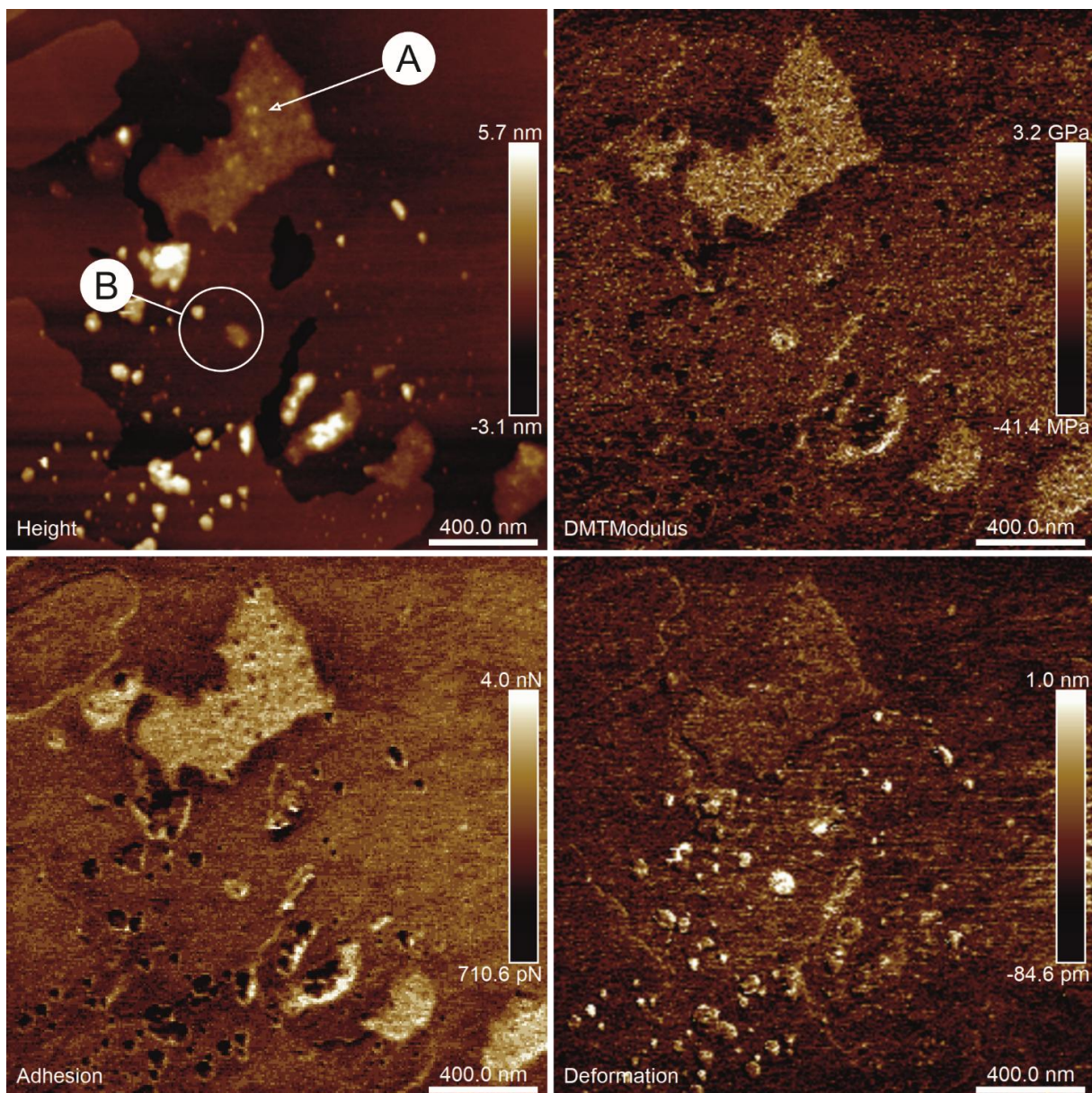

Supplement: S4 Fig — (PDF) [file pone.0229149.s004.pdf]

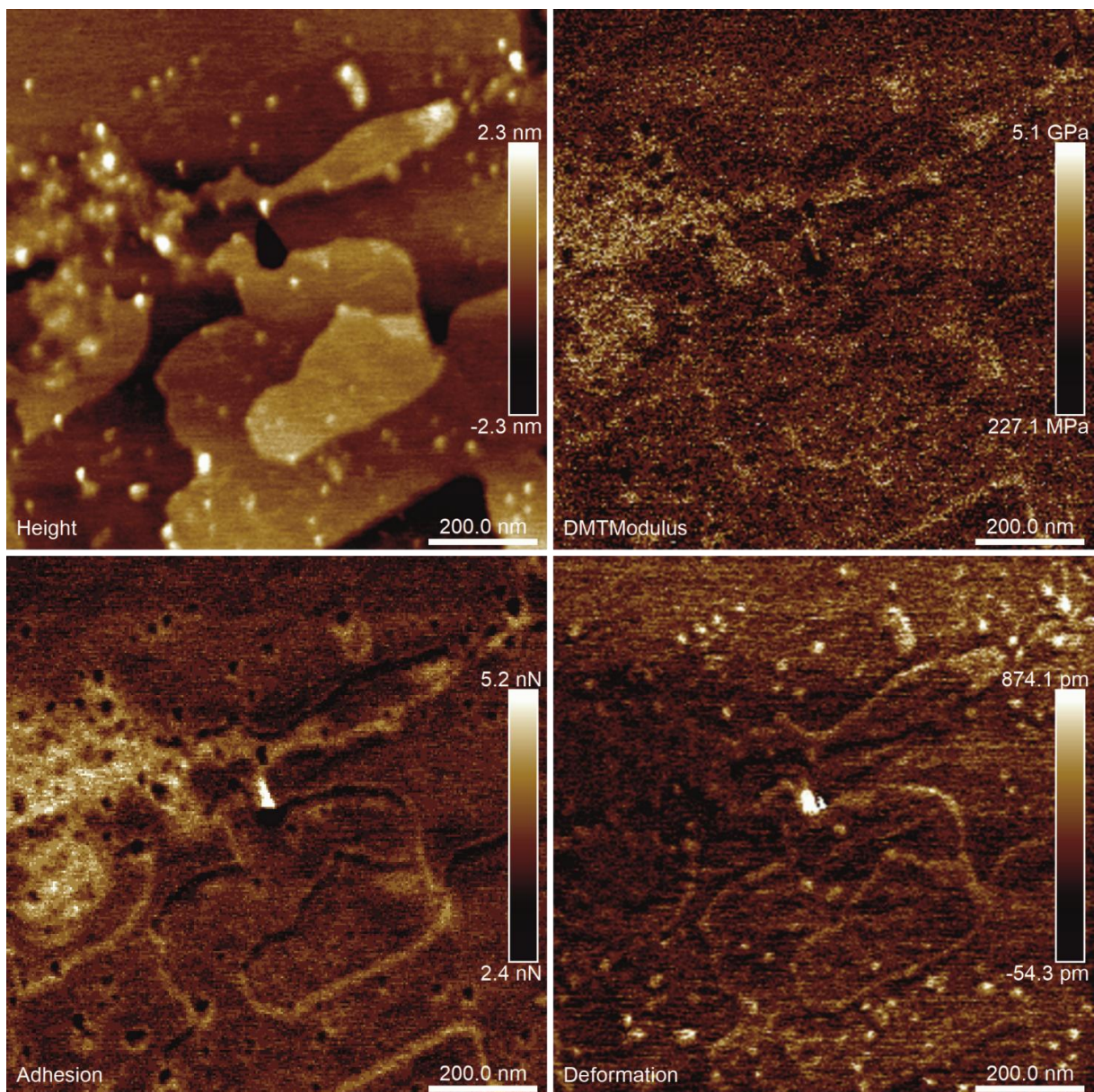

Supplement: S5 Fig — (PDF) [file pone.0229149.s005.pdf]

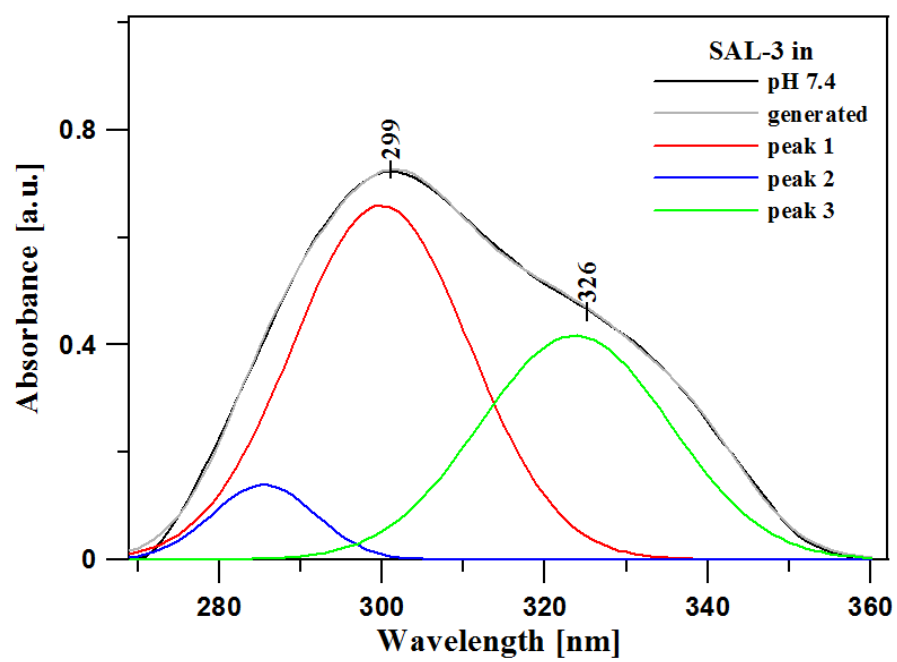

Supplement: S6 Fig — Solid black line–experimental spectrum; solid grey line–spectrum fitted with the decomposition components; blue, red, and green lines–respective components of the given band after decomposition. (PDF) [file pone.0229149.s006.pdf]

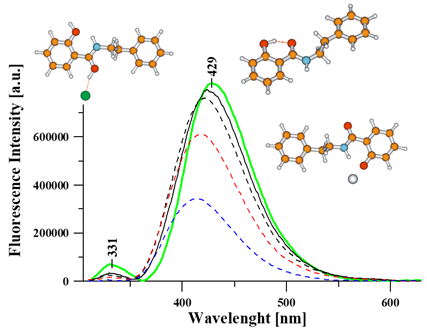

Supplement: S7 Fig — Top–variety of the possible forms of the investigated SAL-3 derivative. Bottom–Fluorescence emission spectra of SAL-3 recorded in H2O : 2-propanol mixture with dual emission marked in green. (PNG) [file pone.0229149.s007.png]
